# Sperm DNA methylation altered by THC and nicotine: Vulnerability of neurodevelopmental genes with bivalent chromatin

**DOI:** 10.1038/s41598-020-72783-0

**Published:** 2020-09-29

**Authors:** Rose Schrott, Maya Rajavel, Kelly Acharya, Zhiqing Huang, Chaitanya Acharya, Andrew Hawkey, Erica Pippen, H. Kim Lyerly, Edward D. Levin, Susan K. Murphy

**Affiliations:** 1 Division of Reproductive Sciences, Department of Obstetrics and Gynecology, Duke University Medical Center, Chesterfield Building, 701 W. Main Street, Suite 510, Durham, NC 27701 USA; 2 Integrated Toxicology and Environmental Health Program, Nicholas School of the Environment, Duke University, Durham, NC USA; 3 Division of Reproductive Endocrinology and Infertility, Department of Obstetrics and Gynecology, Duke University Medical Center, Durham, NC USA; 4 Division of Surgical Sciences, Department of Surgery, Center for Applied Therapeutics, Duke University Medical Center, Durham, NC USA; 5 Department of Psychiatry and Behavioral Sciences, Duke University Medical Center, Durham, NC USA; 6 Department of Pathology, Duke University Medical Center, Durham, NC USA

**Keywords:** DNA methylation, Epigenomics, Spermatogenesis, Autism spectrum disorders

## Abstract

Men consume the most nicotine and cannabis products but impacts on sperm epigenetics are poorly characterized. Evidence suggests that preconception exposure to these drugs alters offspring neurodevelopment. Epigenetics may in part facilitate heritability. We therefore compared effects of exposure to tetrahydrocannabinol (THC) and nicotine on DNA methylation in rat sperm at genes involved in neurodevelopment. Reduced representation bisulfite sequencing data from sperm of rats exposed to THC via oral gavage showed that seven neurodevelopmentally active genes were significantly differentially methylated versus controls. Pyrosequencing data revealed majority overlap in differential methylation in sperm from rats exposed to THC via injection as well as those exposed to nicotine. Neurodevelopmental genes including autism candidates are vulnerable to environmental exposures and common features may mediate this vulnerability. We discovered that autism candidate genes are significantly enriched for bivalent chromatin structure, suggesting this configuration may increase vulnerability of genes in sperm to disrupted methylation.

## Introduction

In the United States (U.S.), rates of autism spectrum disorder (ASD) are climbing. As of 2018, the Centers for Disease Control and Prevention (CDC) reported that 1 in 59 U.S. children are diagnosed with ASD^1^. While the exact cause of ASD remains unknown, it is described as a developmental disorder resulting from interactions between genes and the environment, two major contributors to its multifaceted etiology. No single gene is responsible for ASD; rather about 1000 genes have been identified as candidates that can contribute to the disorder^2^.

Many ASD candidate genes are involved in synaptic growth and regulation, neuronal development, and signaling stability^3^. Alterations of these genes that increase risk of ASD can include copy number variants, single nucleotide polymorphisms (SNPs), mutations, and rare variants^3^. ASD is considered one of the most heritable neurodevelopmental disorders as demonstrated by twin studies and familial studies^3^. However, genetics may not be the only contributing force underlying ASD heritability.

Epigenetic heritability may be another potential mediator of ASD risk. Epigenetics refers primarily to the reversible patterns of histone tail modifications and DNA methylation at CG dinucleotides that contribute to chromatin accessibility and transcription factor binding. These modifications exert a powerful influence on gene regulation and can affect the resulting phenotype without changing the underlying DNA sequence. Epigenetic modifications are a normal and requisite component of developmental processes. However, epigenome alterations can skew spatial and temporal gene expression patterns, leading to phenotypic changes that can contribute to pathology. DNA methylation is the most extensively studied epigenetic regulatory process and plays a critical role in cellular differentiation, DNA compaction inside the nucleus, and regulation of cell-type specific gene expression. The establishment and maintenance of DNA methylation can be influenced by environmental exposures^4^. DNA methylation patterns are normally faithfully reproduced during DNA replication, rendering them somatically heritable. Nutrients, stress, and toxicants are all potentially disruptive to DNA methylation and can result in lifelong perpetuation of altered methylation throughout subsequent cell divisions^4^. This can impact the regulation of gene expression in a manner by which the effects may remain latent unless synergistically combined with other regulatory changes or in a genetic background that favors emergence of a phenotype.

Research has increasingly focused on the role of epigenetics in ASD. Studies have examined post-mortem brains from individuals with ASD, as well as whole blood samples from monozygotic twin pairs discordant for autism and demonstrated differences in DNA methylation in individuals with autism as compared to those without^5,6^. Others have shown that in utero exposure to many substances including polychlorinated biphenyls, pesticides, tobacco smoke, and cannabis are associated with autism and autism-like phenotypes in offspring^7–9^. Associations between autism and early-life exposure to maternal or grandmaternal tobacco smoking during pregnancy have been demonstrated^10,11^. Others have shown associations between maternal cannabis use during pregnancy and neurodevelopmental delay and autistic-like deficits in offspring^12,13^, as well as an increased incidence of autism in offspring exposed to maternal cannabis use during pregnancy^14^. Given that early-life exposure to these different compounds can have similar phenotypic effects in offspring, it is possible that autism candidate genes share an inherent vulnerability to environmental exposures.

The father’s health prior to conception and the exposures he incurs during this time may also impact offspring health and development. Studies show that exposure to cannabis and tobacco products alter sperm DNA methylation^15–17^. There is an urgent need to determine whether these effects are heritable, especially given that men are the predominant cannabis and tobacco product consumers^18–20^, and their use is increasing^18,19^.

The CDC reported in 2016 that 38 million American adults smoked cigarettes “every day” or “some days”^18^. While this number has declined since 2005, rates of cigarette smoking remain high in certain groups. As such, the CDC reports that smoking tobacco cigarettes still remains high among males and among adults aged 25–64^16^. Importantly, this includes adults of reproductive age. Men comprise the majority of recreational cannabis users. A National Survey of Drug Use and Health found in 2018 that 36.9% of American men between ages 18–25 and 16.2% of men over 26 reported using cannabis in the last year, while women in the same age ranges reported 32.6% use and 10.6% use, respectively. When considering past-month use, 11.0% of men over 26 reported using cannabis, while only 6.4% of women of the same age did^21^. Another study that surveyed regular cannabis users found that men use cannabis more frequently and in higher quantities than women^18,19^.

We recently reported on significant differential DNA methylation in sperm of rats exposed to delta-9-tetrahydrocannabinol (THC, the main psychoactive component of cannabis) via oral gavage relative to controls^16^. Oral gavage pharmacokinetically models the consumption of edibles containing THC^16^. Here, we furthered our analysis and identified epigenetic effects of oral gavage THC exposure on genes involved in neurodevelopment. Our list of differentially methylated genes with THC exposure showed significant enrichment of genes involved in neurodevelopment and synaptic plasticity. We were particularly intrigued by this result given our previous finding that cannabis use altered DNA methylation at autism candidate *DLGAP2* in sperm^17^. Turning to individual genes, we focused on seven genes described in the literature as being implicated in autism and autism-like phenotypes^3,22^ and for which sperm DNA methylation was altered by at least 10% following THC exposure via oral gavage. The genes we analyzed are *Dlg4, Shank1, Grid1, Nrxn1, Nrxn3, Syt3,* and *Lrrtm4.* We sought to determine if injection of THC, which pharmacokinetically more closely resembles inhalation, influenced DNA methylation at the same CpG sites in rat sperm. We also examined vulnerability of these genes to other types of exposures by comparing results from the sperm of THC exposed rats to those from sperm of nicotine exposed rats. Lastly, we examined potential commonalities among a known and comprehensive list of autism candidate genes that may make them more vulnerable to exposure-mediated epigenetic alterations.

## Results

### Administration of THC by oral gavage induces differential DNA methylation in sperm at neurodevelopmentally active genes

As previously described, reduced representation bisulfite sequencing (RRBS) data from animals dosed with vehicle (n = 8) or THC by oral gavage (which models oral ingestion of the drug (n = 9) identified 2940 CpG sites (621 genes) that were significantly differentially methylated in the sperm of THC exposed rats compared to controls^16^. This list was entered into the String Database to identify relevant Biological Process Gene Ontology (GO) terms. String recognized 593 genes and generated 166 significant GO terms. We were particularly intrigued by the significant enrichment of genes involved in neurodevelopmental and regulatory processes because of our recent finding that methylation of autism candidate gene *DLGAP2* is significantly altered in sperm from men who use cannabis relative to controls, as well as in the sperm of rats exposed to THC^17^. Furthermore, our results suggested the potential for intergenerational transmission of this methylation change in rats^17^. There were 19 GO terms identified (including 79 unique gene names) that are involved in neuronal development and synaptic plasticity. These included “nervous system development” (p = 2.50E−07 at 5% FDR), “neurogenesis” (p = 1.66E−07 at 5% FDR), “modulation of chemical synaptic transmission” (p = 0.0088 at 5% FDR), and “synapse maturation” (p = 0.028 at 5% FDR). The complete list of all GO terms, with the neuronal-related terms in bold, are included in Table S2.

In addition to the GO terms identified, we focused on seven genes from the initial RRBS dataset that were significantly differentially methylated with a greater than 10% methylation difference and that are known to be involved in neurodevelopmental processes and disorders, including autism. These genes are Discs Large MAGUK Scaffold Protein 4 (*Dlg4*), SH3 and Multiple Ankyrin Repeat Domains 1 (*Shank1*), Glutamate Ionotropic Receptor Delta Type Subunit 1 (*Grid1*), Neurexin 1 (*Nrxn1*), Neurexin 3 (*Nrxn3*), Synaptotagmin 3 (*Syt3*), and Leucine Rich Repeat Transmembrane Neuronal 4 (*Lrrtm4*)*.* THC exposure by oral gavage caused significant hypermethylation at *Lrrtm4* and significant hypomethylation at *Shank1, Syt3, Nrxn1, Nrxn3, Dlg4,* and *Grid1* in rat sperm (Table 1).

**Table 1:** RRBS data.

Chromosome	Gene name	Location	P-value	Methylation difference (%)	Methylation direction
1	*Shank1*	100342877–100342878	0.032	− 11	Hypomethylated
1	*Syt3*	100404328–100404329	0.023	− 11	Hypomethylated
4	*Lrrtm4*	110702815–110702816	0.018	+ 12	Hypermethylated
6	*Nrxn1*	14349149–14349150	0.0064	− 12	Hypomethylated
6	*Nrxn3*	112603748–112603749	0.012	− 11	Hypomethylated
10	*Dlg4*	56638116–56638117	0.048	− 16	Hypomethylated
16	*Grid1*	11321278–11321279	0.020	− 41	Hypomethylated

### THC injection influences differential DNA methylation at neurodevelopmentally active genes

**Figure 1:**
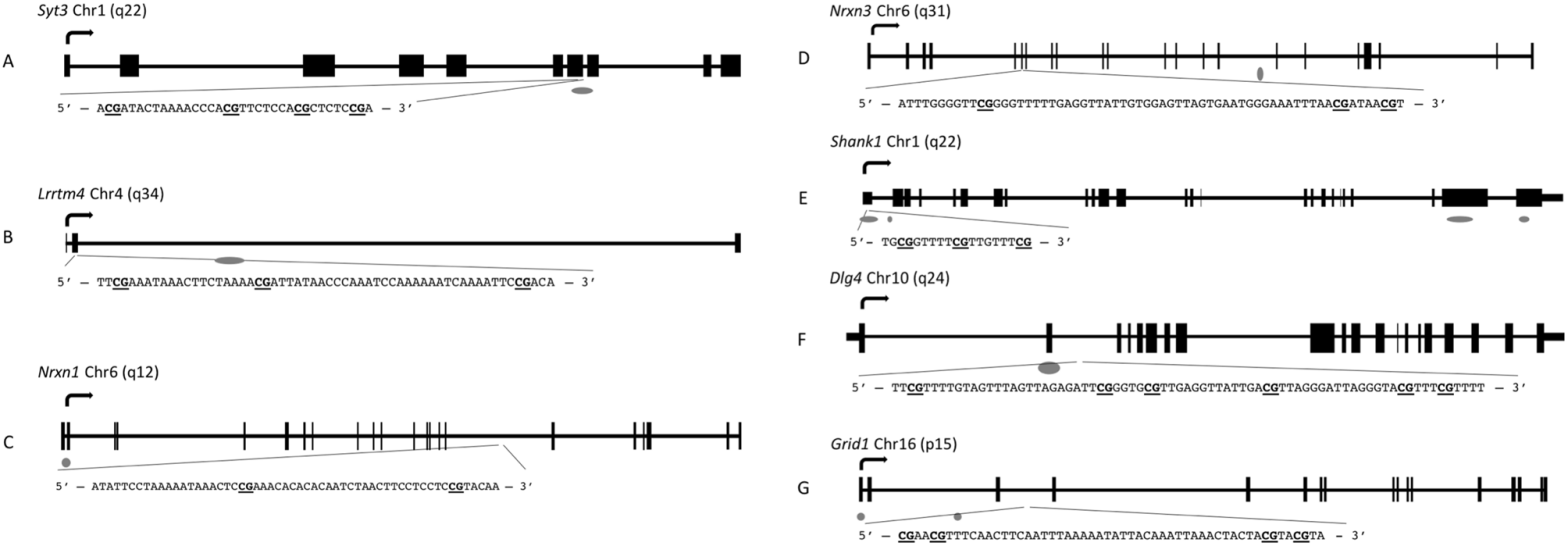
Schematics and sequences analyzed for the seven genes of interest. (**A**) *Syt3*; (**B**) *Lrrtm4*; (**C**) *Nrxn1*; (**D**) *Nrxn3*; (**E**) *Shank1*; (**F**) *Dlg4*; (**G**) *Grid1*. Genes are represented as horizontal lines with rectangles representing exons and grey ovals representing CpG islands. The sequence analyzed by bisulfite pyrosequencing is shown in the insert, with the CG sites in bold and underlined.

**Figure 2:**
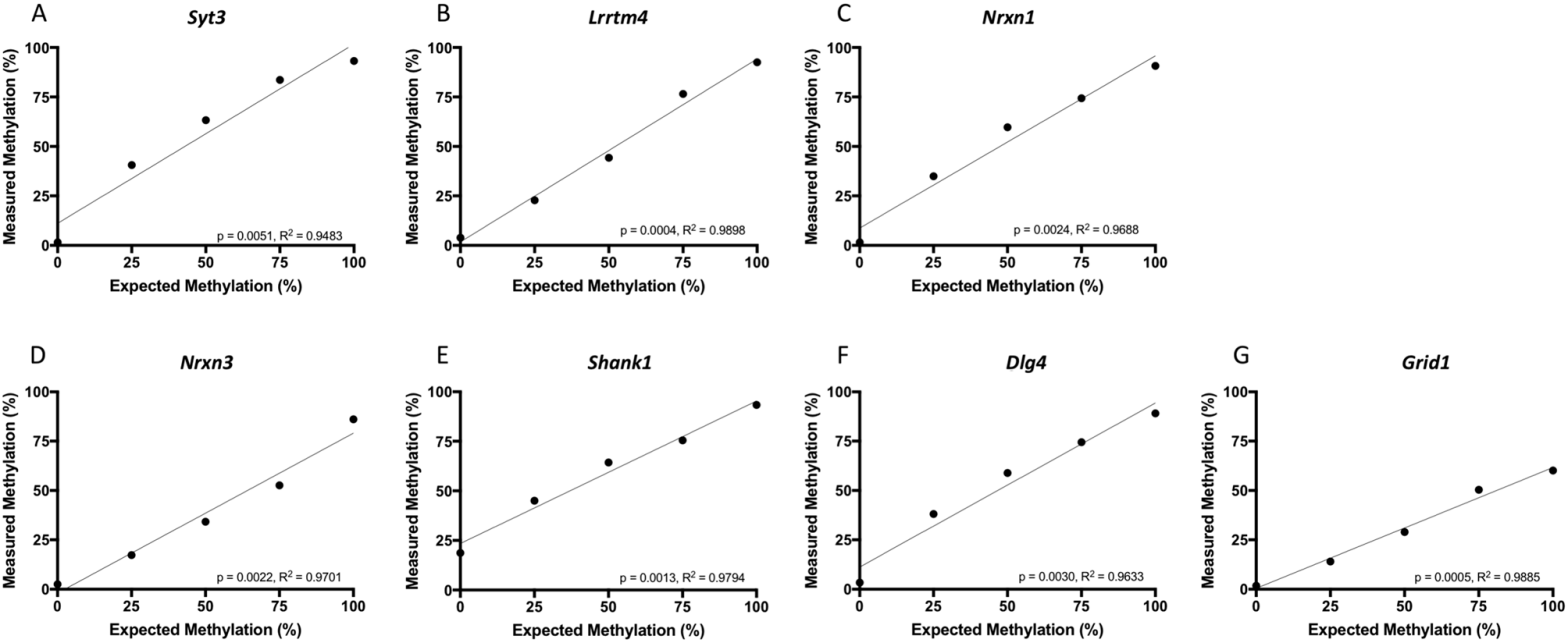
Bisulfite pyrosequencing assay validation. Pyrosequencing assay validation for the seven genes of interest. (**A**) *Syt3*; (**B**) *Lrrtm4*; (**C**) *Nrxn1*; (**D**) *Nrxn3*; (**E**) *Shank1*; (**F**) *Dlg4*; (**G**) *Grid1*. Defined mixtures of bisulfite modified fully methylated and unmethylated rat genomic DNAs were analyzed for linearity in ability to detect increasing amounts of methylation. x-axis, the input (expected) level of methylation; y-axis, the measured level of methylation. R^2^ and p values for Pearson correlation are indicated.

**Figure 3:**
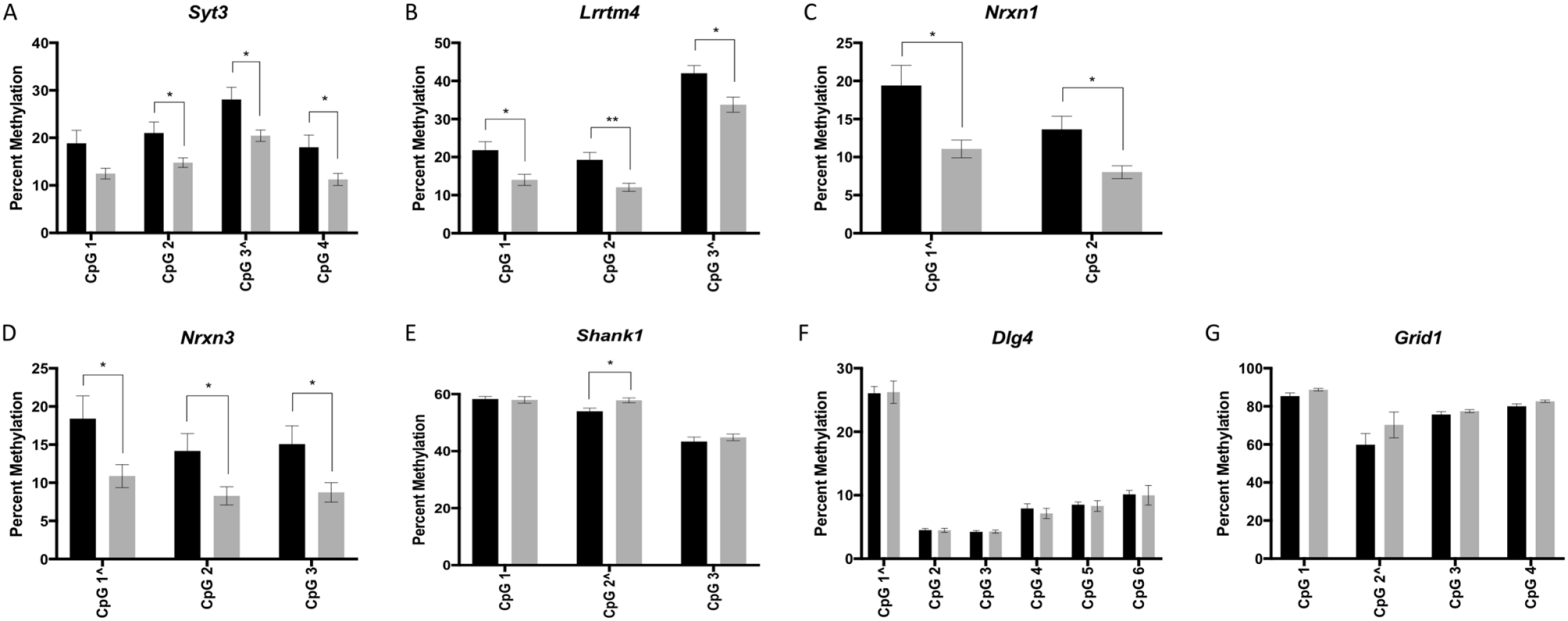
Bisulfite pyrosequencing of sperm DNA from THC injected rats compared to controls. Bar graphs showing bisulfite pyrosequencing results from sperm of THC exposed (gray) versus control (black) rats for (**A**) *Syt3*; (**B**) *Lrrtm4*; (**C**) *Nrxn1*; (**D**) *Nrxn3*; (**E**) *Shank1*; (**F**) *Dlg4*; and (**G**) *Grid1*. Error bars represent the SEM across samples. *p < 0.05; **p < 0.01, unadjusted values. The CpG site labeled with “^” represents the site that was initially identified in the RRBS dataset.

To determine if sperm DNA methylation at the same regions of these genes was similarly affected following subcutaneous injection of THC, quantitative bisulfite pyrosequencing was performed using DNA from sperm of control rats injected with vehicle control (n = 8) or rats injected with 4 mg/kg THC (n = 7), a dose that reflects daily use in humans. Pyrosequencing assays were designed to encompass the same CpG sites identified by RRBS, but also captured neighboring CpG sites (Fig. 1A–G). Assays were validated for performance using defined mixtures of completely methylated and unmethylated bisulfite modified rat DNAs (Fig. 2A–G). Comparing the average methylation across each CpG site for the sperm of exposed versus unexposed rats revealed significant differences: *Syt3, Lrrtm4, Nrxn1, and Nrxn3* were hypomethylated at the CpGs in the region analyzed for each gene (p = 0.009–0.049) while *Shank1* was hypermethylated at the same CpG site that was identified as hypomethylated in the oral gavage studies (p = 0.015) (Fig. 3A–E). *Dlg4* and *Grid1* did not show significant differences in methylation between the exposed and control groups following injection (Fig. 3F,G). The following CpG sites remained significantly affected after Bonferroni correction: CpG site 2 in *Shank1* (adjusted p < 0.013); CpG sites 1, 2, and 3 in *Lrrtm4* (adjusted p < 0.017); and CpG sites 1 and 2 in *Nrxn1* (adjusted p < 0.025).

### Nicotine exposure elicits differential DNA methylation in sperm at neurodevelopmentally active genes

**Figure 4:**
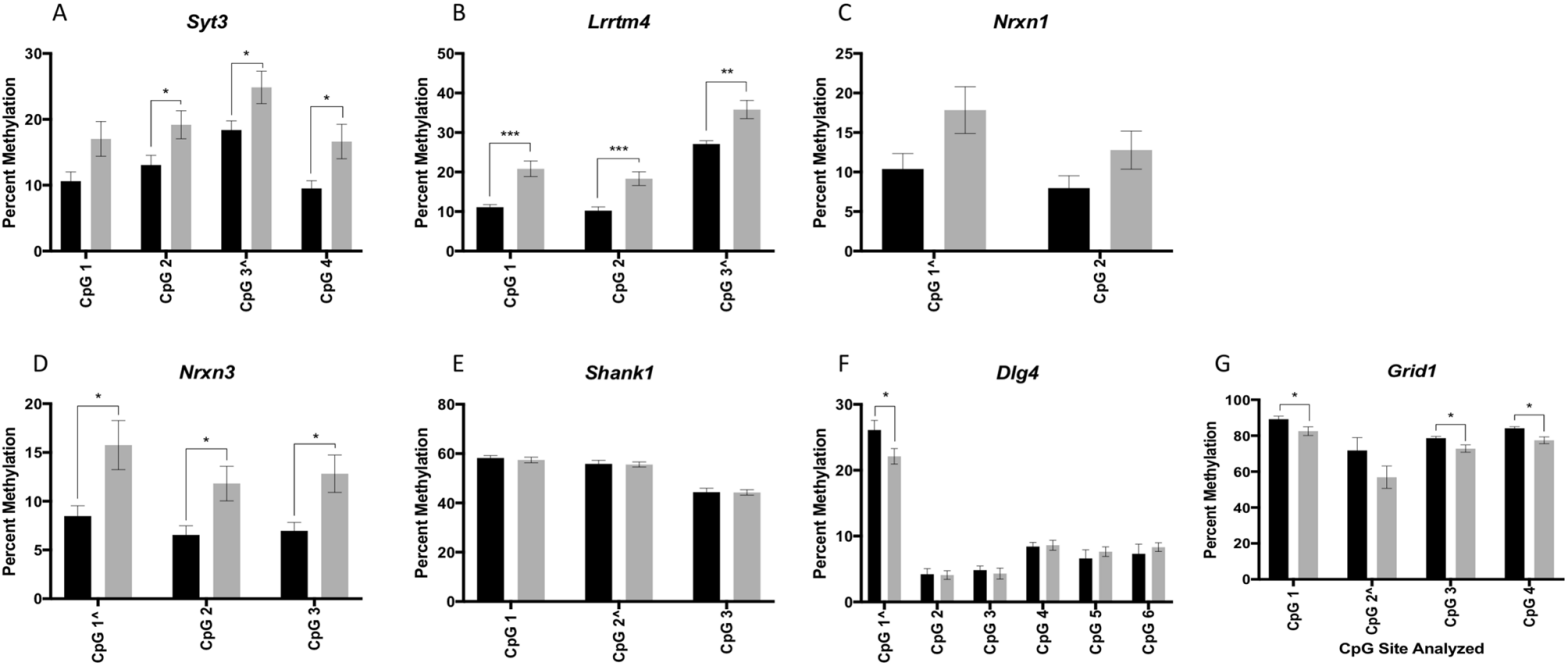
Bisulfite pyrosequencing of sperm DNA from nicotine exposed rats compared to controls. Bar graphs showing bisulfite pyrosequencing results from sperm of nicotine exposed (gray) versus control (black) rats for (**A**) *Syt3*; (**B**) *Lrrtm4*; (**C**) *Nrxn1*; (**D**) *Nrxn3*; (**E**) *Shank1*: (**F**) *Dlg4*; and (**G**) *Grid1*. Error bars represent the SEM across samples. *p < 0.05; **p < 0.01; ***p < 0.005; unadjusted values. The CpG site labeled with “^” represents the site that was initially identified in the RRBS dataset.

Given evidence in the literature describing multiple early life exposures, including cigarette smoke, being associated with offspring autism and autism-like phenotypes, we sought to determine if nicotine, the main neuroactive component of tobacco cigarettes, affected this same subset of genes. Sperm DNA from rats exposed to 2 mg/kg/day nicotine (n = 8) or vehicle control (n = 7) underwent pyrosequencing to compare methylation across each CpG site. Five genes displayed significant differences in methylation in the sperm of nicotine exposed rats as compared to controls: *Syt3, Lrrtm4,* and *Nrxn3* were hypermethylated (p = 0.0007–0.040) in sperm of nicotine exposed rats compared to controls (Fig. 4A,B,D), while *Dlg4* and *Grid1* were hypomethylated in these sperm (p = 0.010–0.050) (Fig. 4F,G). *Nrxn1* and *Shank1* did not exhibit significant differences in methylation (Fig. 4C–E). Following Bonferroni correction, the following CpG sites remained significantly altered: CpG sites 1, 2, and 3 in *Lrrtm4* (adjusted p < 0.017) and CpG site 4 in *Grid1* (adjusted p < 0.017).

### A functional gene network is altered by THC

**Figure 5:**
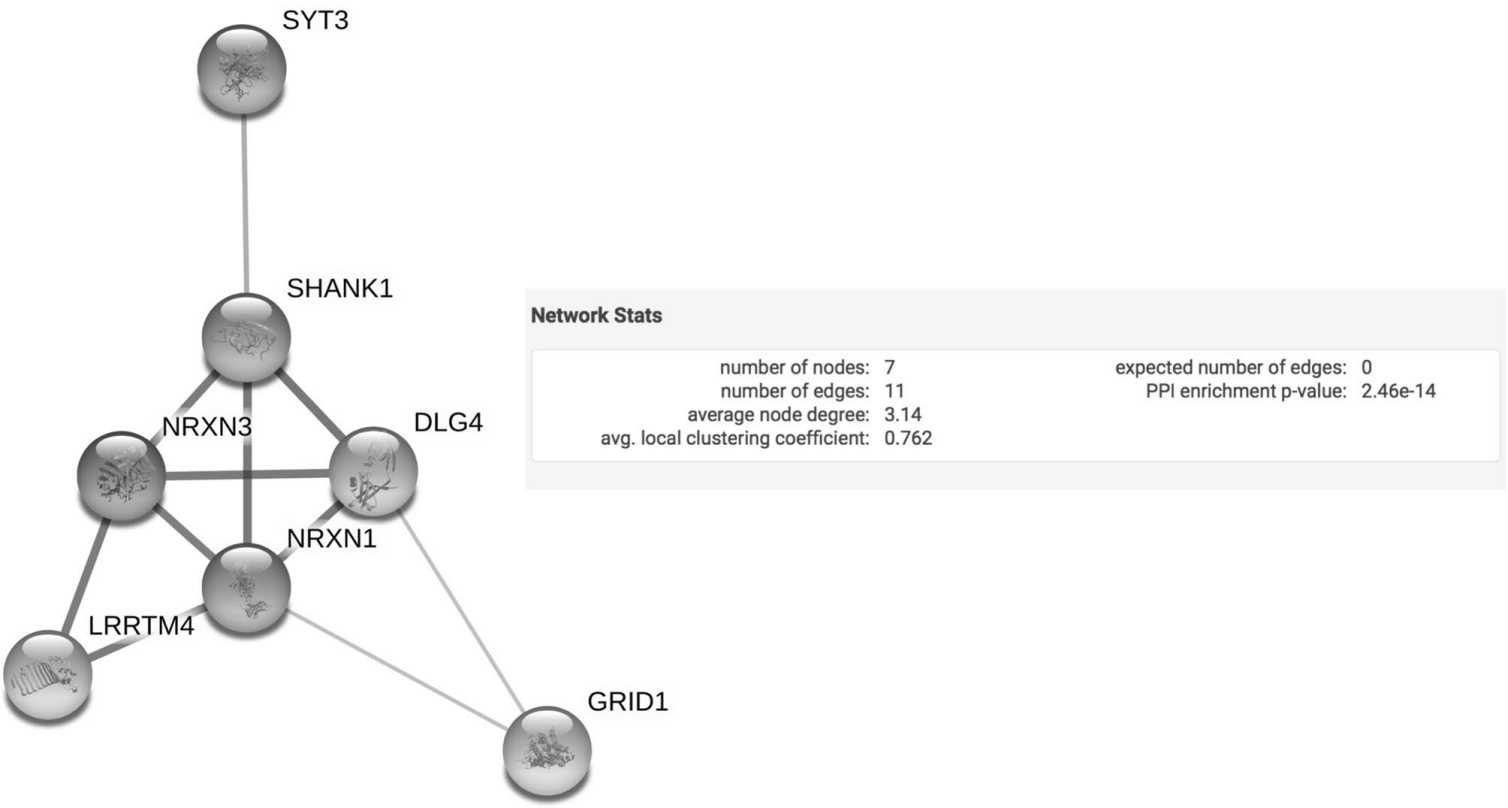
STRING analysis. STRING schematic of protein interactions for the seven genes that were analyzed. The schematic shows that each protein interacts with at least one other protein in this network. The number of nodes represents the number of proteins analyzed, and the number of edges represents the number of interactions present between the seven nodes. The significant PPI enrichment p-value indicates that the interactions of these proteins is not random.

Despite the fact that we knew these genes were each independently implicated in autism, we were curious about whether or not the proteins encoded by these genes actually interact with one another. The seven gene names were evaluated in String to determine if and how they might relate to one another in humans, given the conserved nature of genes implicated in autism^3^ (Fig. 5). This particular group of genes resulted in a significant interaction enrichment value (PPI interaction enrichment p-value, p = 2.46E−14), indicating that the proteins have more functional interactions between each other than expected from a random set of the same number of proteins of similar size from the genome^23,24^. To support that this was not a chance finding, we entered the gene symbols for another independent set of seven control genes (the gene names one position removed from our genes of interest in the spreadsheet of our RRBS results) into String. No significant interaction was detected for these seven control genes (PPI p = 1.0, data not shown). We then chose seven genes at random from the SFARI autism gene list (https://www.sfari.org) and entered them into STRING. We did this a total of ten times, each time with a different randomly chosen group of seven SFARI genes. Of the ten different groups of genes, eight groups had no interactions, one group had only one interaction and one group had three independent interactions. The seven genes studied herein, however, had 11 interactions. The top significant Biological Process GO terms for our seven genes of interest include social behavior (p = 1.70E−09 at 5% FDR), vocalization behavior (p = 3.87E−09 at 5% FDR), and learning (p = 9.93E−06 at 5% FDR) (Table S3). Social and communication deficits are core affected domains in autism, and these deficits often impact learning.

### Autism candidate genes are enriched with bivalent chromatin marks

**Figure 6:**
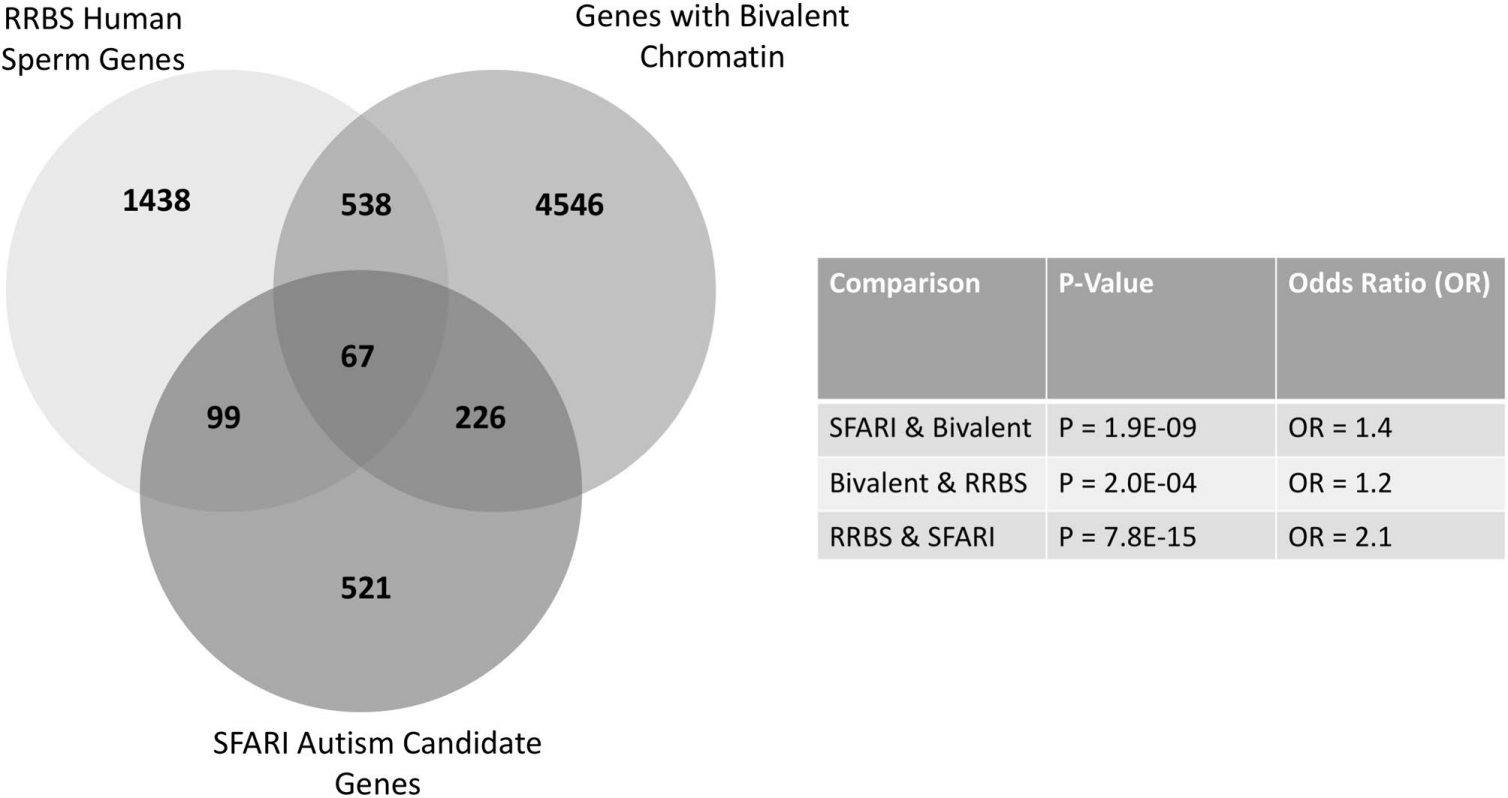
Significant overlap between SFARI autism genes, genes with bivalent chromatin, and genes from human RRBS study that are differentially methylated in sperm between cannabis users and non-user controls. Venn diagram showing the number of genes that are included on the SFARI autism candidate gene list, the list of genes having bivalent chromatin marks in human embryonic stem cells, and those that were identified as being significantly differentially methylated in human sperm of men who used cannabis compared to those who did not. There is significant overlap (p < 0.05) when comparing SFARI to bivalent, SFARI to RRBS, and bivalent to RBBS genes. An odds ratio > 1 demonstrates a strong relationship between lists.

We observed significant differences in sperm DNA methylation following THC and nicotine exposure at an overlapping group of neurodevelopmentally important genes and the literature describes multiple exposures as being implicated in autism and autism-like phenotypes^7,9,25^. The abundance of genes that are important mediators of early growth and development identified by others^26–29^ and our group^16,17,30–34^ that exhibit altered methylation resulting from environmental exposures suggests they may share common features that render them vulnerable. Many genes that are involved in early development require activation in a highly regulated temporal manner and are associated with the presence of bivalent chromatin marks. We therefore hypothesized that the bivalent status of genes may make them more epigenetically vulnerable to environmental exposures. Using two publicly available datasets, we identified 226 overlapping genes between the list of 913 autism candidate genes from SFARI and 5,377 genes that possess bivalent chromatin marks in human embryonic stem cells as identified by Court and Arnaud^2,35^. This overlap is statistically significant (Fig. 6, p = 1.9E−09, Odds ratio = 1.4) and suggests that a large number of autism candidate genes possess bivalent chromatin markings. We then looked for potential overlap between genes with bivalent chromatin and our RRBS list of genes in human sperm that were significantly differentially methylated in cannabis users compared to non-user controls. We found that there are 538 genes in common between the two lists of genes. This too was statistically significant (Fig. 6, p = 2.0E−04, Odds ratio = 1.2). Lastly, we compared the list of SFARI genes to the human RRBS genes and found 99 genes in common, a statically significant overlap (Fig. 6, p = 7.8E−15, Odds ratio = 2.1). There were 67 genes in common among all three lists. These results support our hypothesis that bivalent chromatin structure makes genes inherently vulnerable to disruption of DNA methylation and potentially altered expression as a result of environmental exposures. Of interest, of the seven genes that we examined, four of them are found in both the SFARI gene list and also have bivalent chromatin marks.

## Discussion

We examined the effects of THC exposure by oral gavage or injection and nicotine exposure on DNA methylation in rat sperm. Our focus was on seven genes implicated in autism. While genome-wide effects have been identified in human exposure studies of cannabis and tobacco cigarettes as well as in rodent models, fewer studies are focused on functionally related groups of genes, adding to the novelty of our findings. We interrogated the RRBS dataset and analyzed gene ontology terms associated with the differentially methylated genes. We were surprised to find that a large number were implicated in neurodevelopment and neuronal processes. Given our previous demonstration that paternal cannabis use is associated with altered methylation of autism candidate *DLGAP2* and our findings of effects on genes involved in neurodevelopment^17^, we focused on genes previously reported in the literature to play a role in autism and for which there was a greater than 10% difference in sperm DNA methylation following exposure to 2 mg/kg THC via oral gavage as compared to vehicle-exposed controls. Seven genes met these criteria, including discs large MAGUK scaffold protein 4 (*Dlg4*)*,* SH3 and multiple ankyrin repeat domains 1 (*Shank1*), glutamate ionotropic receptor delta type subunit 1 (*Grid1*)*,* neurexin 1 (*Nrxn1*)*,* neurexin 3 (*Nrxn3*)*,* synaptotagmin 3 (*Syt3*)*,* and leucine rich repeat transmembrane neuronal 4 (*Lrrtm4*). The route of THC exposure models oral consumption at a dose pharmacodynamically equivalent to moderate human consumption^36^.

We wanted to determine whether the effects of THC on methylation at these seven genes were independent of route of exposure. Injection of 4 mg/kg THC models inhalation at a dose pharmacodynamically equivalent to heavier human consumption^36^. Using quantitative bisulfite pyrosequencing, we observed a significant difference in methylation at five of the seven genes that were originally identified in sperm following oral gavage of THC, with *Dlg4* and *Grid1* showing no significant differences. Reasons for inability to validate two of the genes may be due to small sample size, inadequate read depth for these regions in RRBS, or bias in amplification prior to RRBS. Interestingly, there was a difference in the direction of methylation change between the two routes of exposure at *Shank1* and *Lrrtm4*, which might be attributable to route-specific effects. Nevertheless, these results highlight that different routes of THC exposure can affect DNA methylation changes at genes important for neurodevelopment.

We determined that these same THC-vulnerable genes were also susceptible to nicotine exposure. We analyzed DNA methylation in sperm of rats exposed to 2 mg/kg nicotine, a dose mimicking moderate tobacco smoking in humans. Pyrosequencing of sperm showed significant differences in DNA methylation at five of the seven genes, with *Shank1* and *Nrxn1* showing no significant differences at the CpG dinucleotides covered by our assays. Interestingly, the direction of change of methylation was opposite between injected THC and nicotine at *Syt3, Lrrtm4,* and *Nrxn3*. *Dlg4* and *Grid1* were significantly hypomethylated in exposed rat sperm with both nicotine and oral THC while they were not significantly altered with injected THC. We were surprised to see these discrepancies, especially between the two routes of THC exposure, as well as the differences in direction of methylation between THC and nicotine at certain genes. We don’t have a complete understanding of these effects, but what is interesting is the specificity of the effects that we observed. Genes may exhibit a general vulnerability to exposures, but the epigenetic consequence itself may in some cases be exposure-dependent, as suggested from our results. It is important to note that the experimental exposures of the male rats to nicotine and THC were each conducted under controlled conditions as single exposures. In reality, we are exposed to broad mixtures of compounds each day. Therefore, we ultimately need to move to better understand the combinatorial effects of these exposures on DNA methylation. Our findings presented here, however, prompted us to further investigate why these particular genes are targeted.

Using the String database to examine functional relationships between the proteins produced by these genes, the top Biological Process GO terms identified were social behavior, vocalization behavior, and learning. Autism spectrum disorders are defined as complex neurodevelopmental disorders characterized by impairments in social interactions, language and communication which often affect learning^37^. Further, we found that the proteins encoded by these genes are highly enriched for functional interactions with each other. Our findings underscore the need for additional studies to better understand how paternal preconception exposures might contribute to the development of neurologic disorders like ASD.

*Dlg4, Shank1, Grid1, Nrxn1, Nrxn3, Syt3,* and *Lrrtm4* are all genetically implicated in autism^3^. Mutations in *Shank1, Nrxn1, Nrxn3,* and *Grid1* have been identified in individuals with the disorder^3,6,22,38^, while SNPs in *Dlg4* have been linked to autism etiologies^3^. Copy number variations leading to deletions of the last three exons of *Shank1* and the entirety of the *Syt3* gene were identified in a cohort of individuals with ASD in Europe, and variants of *Lrrtm4* have been reported in ASD^22,38^. Recent studies have also begun to focus on epigenetic regulation of autism candidate genes and the role of the environment in disrupting epigenetic regulation in autism etiology^39,40^. The role of epigenetic “writers”, including DNA methyltransferase enzymes, has been studied in autism, where mutations in *DNMT3A* have been detected in ASD cases^41^. Additionally, epigenetic dysregulation of individual autism candidate genes has been demonstrated in peripheral blood and brain tissues of individuals with autism^42^. *Nrxn1* DNA methylation changes have been correlated with social autistic trait scores in human autism cohorts, demonstrating an example of a gene that is both genetically and epigenetically implicated in ASD^6^. It will be important to determine if genetic alterations are mutually exclusive with DNA methylation alterations within the same individual at the same gene, as has been found in the majority of studies at the *BRCA1* and *RAD51* loci in cancers^43–45^.

Epigenetic changes can alter a gene’s expression, and small methylation changes can have significant effects on expression^46^. This is particularly true for genes that play an acute role in neuronal processes that are precisely controlled. For example, *Shank1* and *Dlg4*, both located in the post-synaptic density (PSD) of neurons, play a critical role in regulating synaptic scaling and plasticity, a tightly regulated process that can have severe consequences when disrupted^47,48^. As such, changes in the expression of PSD genes have been associated with disorders such as autism and schizophrenia^47–49^. We recently demonstrated that methylation of another PSD gene, *DLGAP2*^50^, is significantly altered in the sperm of human cannabis users as compared to controls^17^. We also showed that *Dlgap2* was hypomethylated in sperm of THC exposed rats compared to controls, and that this change persisted in the nucleus accumbens of pups born to THC exposed fathers^17^. *Dlgap2* has also been shown by others to be differentially methylated in the nucleus accumbens of rats born to parents with adolescent THC exposure^51^. We found that methylation of *DLGAP2* in human brain tissue at the same region altered in sperm was inversely related to expression of this gene, supporting that altered DNA methylation has functional consequences for the levels of gene product produced^17^.

We previously published that cannabis use is associated with methylation alterations in a large number of genes important for early development^16^. Given that multiple in utero and early-life exposures are associated with autism and autism-like phenotypes, and our findings that two different exposures elicited methylation changes at autism candidate genes in sperm, we questioned whether there was something about autism candidate genes and early development that might make the epigenetic information these genes normally contain inherently vulnerable to environmental exposures. Bivalent chromatin is epigenetically marked by both active (H3K4me3) and repressive histone (H3K27me3) marks^52^. These dual markings help keep genes silent but poises them for rapid activation when triggered by developmental cues early in life. As such, bivalent chromatin characterizes many genes critical for early development^52^. The presence of DNA methylation has been shown to coincide with regions of the genome that possess bivalent chromatin, specifically at CpG islands^53^. Therefore, the chromatin state formed by one epigenetic modification (e.g. bivalent chromatin) could render another epigenetic modification (e.g. DNA methylation) more vulnerable to disruption by the environment. Indeed, age-associated changes in DNA methylation have been reported to occur preferentially at bivalently marked domains^54^ and bivalent chromatin may also make tumor suppressor genes vulnerable to hypermethylation in cancer^55^. Supporting our contention that bivalent chromatin increases epigenetic vulnerability of neurodevelopmentally important genes, studies have shown that DNA methylation at a subset of bivalently poised loci is aberrant in post-mortem brain samples of autistic individuals^56^. However, the enhanced vulnerability of DNA methylation at these loci to environmental exposures requires further focused study. It was striking to discover there was a significant overlap between a known list of autism candidate genes and a list of genes with bivalent chromatin. This finding begins to support our hypothesis that bivalent architecture enhances the vulnerability of autism candidate genes to perturbations by multiple environmental exposures. The significant overlap between our human RRBS gene list with both the list of genes that possess bivalent chromatin and the SFARI autism gene list further supports this hypothesis and provides a strong foundation for future studies in this area. It will be critical for follow up studies to determine whether or not the associations with specific CpG sites correlate with areas of the gene that are marked with bivalent chromatin.

While about 90% of histone proteins in sperm are replaced with highly specialized and sperm-specific protamines, the histones that are retained indeed carry functional significance^57^. Interestingly, bivalent chromatin markings are frequently found at retained histones in sperm^57^. Further, developmental loci that possess bivalent marks in sperm are also found to be bivalently marked in the early embryo^57–59^. This correlation suggests that these epigenetic marks present in the early embryo may be transmitted from the paternal germline, supporting a possible route of epigenetic intergenerational inheritance^58^.

Men in the U.S. are the predominant users of both cannabis and tobacco products. Despite efforts to promote cigarette smoking cessation, cigarette use among men still remains high, and the use of electronic delivery systems is rapidly expanding^18^. While tobacco products have been legal for those 18 years and older in the U.S. for decades, consumption of cannabis products is only recently undergoing expanding legalization. As a result, cannabis use is increasing and is coinciding with an increase in the percentage of the public that believes cannabis use is safe^60^. Male cannabis use has been associated with reduced fertility and in most^61^ but not all^62^ studies, decreased sperm counts. Initial epigenetic studies by our group have shown that male cannabis use, and male rat exposure to THC, cause widespread DNA methylation changes in sperm^16^. Furthermore, others have shown that cigarette smoking significantly alters DNA methylation patterns in male sperm^63,64^, showing that there are also genome-wide epigenetic effects of this exposure. The significance and impact of these changes in sperm on offspring health and development are only beginning to be explored—yet results may provide important information and critical opportunities for preventive interventions to reduce risk to future children.

Our results provide new information about the effects of THC exposure by multiple routes, and of nicotine exposure on the DNA methylation status of a group of genes implicated in autism. While rates of this complex neurodevelopmental disorder are increasing in the U.S., the cause of this rise remains unknown. Studies of exposure to cigarette smoke are increasingly pointing to the need to consider paternal smoking history in the context of offspring autism and autism-like phenotypes^65^. It will also be important to consider the possibility of multi-generational transmission of epigenetic alterations, which may be more likely if they are occurring at bivalently poised (and histone-retaining) sites throughout the sperm genome. If true, it is possible the increase in diagnoses may in part reflect the widespread use of tobacco cigarettes in past generations, particularly since cigarette use peaked in the early 1960s^66^. In this regard, it is interesting to note that the rapid rise in autism diagnoses began between 1985 and 1995, in children born to adults of whom > 40% were themselves exposed to tobacco smoke preconceptionally^66^. In utero exposure of these adults would have also simultaneously exposed the developing gametes of the subsequent generation, and it is in this subsequent generation that autism prevalence began to rise. With respect to the increasing prevalence of cannabis use, Reece and Hulse reported that autism is the most common form of cannabis-associated clinical teratology in the U.S. Their statistical models projected that by the year 2030, states with some form of legal cannabis would have a 60% excess of autism cases compared to those states that have not legalized cannabis^12^.

Our study has several limitations. The sample size was relatively small which may have hindered our ability to reveal significant differences at all genes across all three exposure paradigms assessed. However, despite our small sample size, we were able to detect a large number of significant differences at the genes analyzed in sperm, which supports the specificity of exposure effects. Additional studies are needed to confirm findings. While we were able to characterize these changes in the sperm of exposed rats, we have not yet examined potential heritability of these changes. Initial studies have demonstrated behavioral effects as the result of paternal exposure to nicotine^67^ and THC^68^. It will be important to examine offspring brain DNA methylation and relationship to behavioral effects. Lastly, there were different durations of the exposures, with only the nicotine exposure spanning the entirety of rat spermatogenesis. We identified significant differences at all durations of exposure, indicating that the changes in DNA methylation are able to occur minimally in maturing or mature sperm. However, longer duration exposure may enhance the ability to detect additional differences that may be occurring in the spermatogonia.

Study strengths include the use of two different sequencing methodologies to identify and analyze DNA methylation and targeted regions of the genome. This study was the first to demonstrate that a distinct group of genes associated with autism are significantly differentially methylated in the sperm of rats exposed to THC and nicotine. This highlights the potential vulnerability of this particular group of genes in sperm. Furthermore, this is the first demonstration of a significant overlap between a comprehensive list of known autism candidate genes and genes with bivalent chromatin structure. Cannabis and tobacco use are highest among men of reproductive age, and while use is increasing, the potential health implications need more intensive examination. More research is needed to improve understanding of how cannabis and tobacco use impact the sperm epigenome and whether those impacts are heritable.

## Methods

All animal study protocols were approved by the Institutional Animal Care and Use Committee at Duke University and conducted in accordance with federal guidelines.

### Rat THC exposure via oral gavage

Young adult, sexually mature male Sprague Dawley rats were purchased from Charles River Laboratories, housed 2–3 per cage, and were dosed daily for 12 days via oral gavage with either 4 ml vehicle control (n = 8, 10% ethanol, 1% Triton X-100 in saline) or 2 mg/kg THC in 1% Triton X-100 in saline (n = 9, dose models moderate human use^36^) (Sigma-Aldrich St Louis, MO, USA) as described previously^16^. Rats were sacrificed and the epididymis was placed in sterile PBS where the swim out method enriched the solution for mature (motile) sperm. Sperm were washed with PBS and were frozen before being transferred to -80 °C for further use.

### Rat THC exposure via injection

Young adult, sexually mature male Sprague–Dawley rats were purchased from Charles River Laboratories and were housed 2–3 per cage. Rats were randomized to two groups that were dosed daily for 28 days via subcutaneous injection with vehicle only (4% TWEEN-80 in saline, n = 8) or 4 mg/kg THC (models heavy human use^36^, n = 7) as described previously^17^. Following exposure, sperm was collected and stored as described above.

### Rat nicotine exposure

Male rats were exposed to nicotine (Sigma Aldrich, St. Louis, MO, USA) as described by Hawkey et al. 2019^67^. Briefly, chronic exposure to nicotine detartrate was delivered via osmotic minipump (Alzet model 2ML4, Durect Inc., Cupertino, CA, USA) at 2 mg/kg/day (dose calculated as of the nicotine base weight, n = 8). Minipumps delivered consistent exposure for 28 days, with two consecutive minipumps implanted on opposite flanks of the body for a total duration of 56 days. Controls (n = 7) received the same surgery and pumps, but pumps contained only the saline vehicle. Following exposure, sperm was collected and stored as described above.

### Human participants

Participants (ages 18–40) were screened and recruited as previously described by Murphy et al. 2018, resulting in 12 cannabis users and 12 non-user controls enrolled in the study^16^. All study procedures were reviewed and approved by the Duke Institutional Review Board and were conducted in accordance with the 2013 Declaration of Helsinki. Written informed consent was provided by all participants.

### DNA isolation from sperm

DNA extraction was performed as described in Schrott et al. 2019^17^. Briefly, DNA was extracted from rat and human sperm samples according to Qiagen’s Puregene DNA Purification Protocol (Qiagen, Germantown, MD, USA). Sperm samples underwent cell lysis and Proteinase K digestion, followed by treatment with RNase A solution. Following protein precipitation and removal, DNA was isolated and eluted in 30 μl of nuclease-free water. Genomic DNA (gDNA) was quantified on the NanoDrop 2000 and quality was determined through measurement of the A_260/280_ and A_260/230_ ratios. gDNA was stored at − 20 °C for subsequent studies.

### Reduced representation bisulfite sequencing (RRBS) 

Genomic DNA from sperm of rats receiving THC/vehicle by oral gavage, as well as from the sperm of 12 cannabis users and 12 non-user controls, was used for RRBS. Full details of the methodology and analytical approach are available in Murphy et al. 2018^16^. RRBS sequencing metrics are included in Table S4. Rat RRBS data are available at the NCBI Sequence Read Archive under accession number PRJNA633085. Human RRBS data is available from the Duke Research Data Repository at URL: 10.7924/r4v122j79.

### Identification of neurodevelopmental genes significantly affected in sperm of oral gavage exposed rats

Following data analysis, significantly differentially methylated CpG sites that met all criteria were annotated to genes (described in Murphy et al. 2018^16^) and these unique gene names were then entered into the String Database (string-db.org). Genes from significantly enriched biological process GO terms were interrogated and a group of seven genes were chosen for subsequent analysis given (1) their role in neurodevelopmental processes, (2) their described roles in the literature in autism^3,6,22,38^, and (3) their greater than 10% difference in sperm DNA methylation between the exposed and control male rats.

### Bisulfite conversion, polymerase chain reaction (PCR) and bisulfite pyrosequencing

The column-based EZ DNA Methylation kit (Zymo Research; Irvine, CA, USA) was used to treat 800 ng gDNA with sodium bisulfite to convert all unmethylated cytosinces to uracils, (that ultimately appear as thymines following downstream PCR and sequencing), while allowing all methylated cytosines to remain cytosines in the sequence. This resulted in bisulfite modified DNA (bsDNA) at a final concentration of 20 ng/μl. bsDNA (40 ng) was then used as a template for PCR amplification for bisulfite pyrosequencing. Bisulfite pyrosequencing assay design, validation, and sequencing were performed as described previously^69^. Primers and PCR conditions are listed in Table S1.

### Statistical analyses

Statistics were performed in GraphPad Prism Version 8 (GraphPad Software, San Diego, CA, USA) and R Studio R Package for Testing and Visualizing Gene Overlaps^70^. For pyrosequencing assay validation, Pearson correlations determined the relationship between the input and measured amounts of DNA methylation and significance. For bisulfite pyrosequencing, a two-tailed Student’s *t* test was run for each CpG site, comparing the means of the exposed to the unexposed rats. Unadjusted p-values and those that remained significant after Bonferroni correction for the number of CpG sites analyzed are reported. To compare overlap between the SFARI, bivalent, and human RRBS gene lists, a Fisher’s exact test was used to determine significance and odds ratios. P values < 0.05 were considered significant and an odds ratio > 1 suggests the association between lists is strong.

## Supplementary information

Supplementary Tables.

## Data Availability

Data is available from the authors upon request. Rat RRBS data is available from the NCBI Sequence Read Archive, accession number PRJNA633085. Human RRBS data is available from the Duke Research Data Repository under Creative Commons CC0 1.0 Universal rights, which is accessible at the following URL: https://doi.org/10.7924/r4v122j79.

## References

[1] Centers for Disease Control and Prevention. *Data and Statistics on Autism Spectrum Disorder*. https://www.cdc.gov/ncbddd/autism/data.html (2019).

[2] Simons Foundation Autism Research Initiative. *SFARI Human Gene Module*. https://www.gene.sfari.org (2019).

[3] Banerjee S, Riordan M, Bhat MA (2014). Genetic aspects of autism spectrum disorders: insights from animal models. Front. Cell Neurosci..

[4] Dolinoy DC, Jirtle RL (2008). Environmental epigenomics in human health and disease. Environ. Mol. Mutagen.

[5] Nardone S (2014). DNA methylation analysis of the autistic brain reveals multiple dysregulated biological pathways. Transl. Psychiatry.

[6] Wong CC (2014). Methylomic analysis of monozygotic twins discordant for autism spectrum disorder and related behavioural traits. Mol. Psychiatry.

[7] Sealey LA (2016). Environmental factors in the development of autism spectrum disorders. Environ. Int..

[8] Ye BS, Leung AOW, Wong MH (2017). The association of environmental toxicants and autism spectrum disorders in children. Environ. Pollut..

[9] Kalkbrenner AE, Schmidt RJ, Penlesky AC (2014). Environmental chemical exposures and autism spectrum disorders: A review of the epidemiological evidence. Curr. Probl. Pediatr. Adolesc. Health Care.

[10] Golding J (2017). Grand-maternal smoking in pregnancy and grandchild's autistic traits and diagnosed autism. Sci. Rep..

[11] Chatterton Z (2017). In utero exposure to maternal smoking is associated with DNA methylation alterations and reduced neuronal content in the developing fetal brain. Epigenet. Chromatin.

[12] Reece AS, Hulse GK (2019). Effect of cannabis legalization on US autism incidence and medium term projections. Clin. Pediatr. Open Access.

[13] Reece AS, Hulse GK (2019). Epidemiological associations of various substances and multiple cannabinoids with autism in USA. Clin. Pediatr. Open Access.

[14] Corsi DJ (2020). Maternal cannabis use in pregnancy and child neurodevelopmental outcomes. Nat. Med..

[15] Morkve Knudsen GT (2019). Epigenome-wide association of father's smoking with offspring DNA methylation: A hypothesis-generating study. Environ. Epigenet..

[16] Murphy SK (2018). Cannabinoid exposure and altered DNA methylation in rat and human sperm. Epigenetics.

[17] Schrott R (2019). Cannabis use is associated with potentially heritable widespread changes in autism candidate gene DLGAP2 DNA methylation in sperm. Epigenetics.

[18] Centers for Disease Control and Prevention. *Smoking is down, but almost 38 million American adults still smoke*. https://www.cdc.gov/media/releases/2018/p0118-smoking-rates-declining-infographic.html (2018).

[19] Cuttler C, Mischley LK, Sexton M (2016). Sex differences in cannabis use and effects: A cross-sectional survey of cannabis users. Cannabis Cannabinoid Res..

[20] National Institute on Drug Abuse. *Sex and Gender Differences in Substance Use.* https://www.drugabuse.gov/publications/research-reports/substance-use-in-women/sex-gender-differences-in-substance-use (2018).

[21] Substance Abuse and Mental Health Services Administration. *2018 National Survey of Drug Use and Health *(*NSDUH*)* Releases*. https://www.samhsa.gov/data/release/2018-national-survey-drug-use-and-health-nsduh-releases (2018).

[22] de Wit J, Ghosh A (2014). Control of neural circuit formation by leucine-rich repeat proteins. Trends Neurosci..

[23] Szklarczyk D (2019). STRING v11: Protein-protein association networks with increased coverage, supporting functional discovery in genome-wide experimental datasets. Nucleic Acids Res..

[24] Szklarczyk D (2017). The STRING database in 2017: Quality-controlled protein–protein association networks, made broadly accessible. Nucleic Acids Res..

[25] Keil KP, Lein PJ (2016). DNA methylation: A mechanism linking environmental chemical exposures to risk of autism spectrum disorders?. Environ. Epigenet..

[26] Perera F, Herbstman J (2011). Prenatal environmental exposures, epigenetics, and disease. Reprod. Toxicol..

[27] Vaiserman A (2015). Epidemiologic evidence for association between adverse environmental exposures in early life and epigenetic variation: A potential link to disease susceptibility?. Clin. Epigenet..

[28] Carvan MJ (2017). Mercury-induced epigenetic transgenerational inheritance of abnormal neurobehavior is correlated with sperm epimutations in zebrafish. PLoS ONE.

[29] Herbstman JB (2010). Prenatal exposure to PBDEs and neurodevelopment. Environ. Health Perspect..

[30] Soubry A (2013). Paternal obesity is associated with *IGF2* hypomethylation in newborns: Results from a Newborn Epigenetics Study (NEST) cohort. BMC Med..

[31] Joubert BR (2012). 450K epigenome-wide scan identifies differential DNA methylation in newborns related to maternal smoking during pregnancy. Environ. Health Perspect..

[32] Soubry A (2015). Newborns of obese parents have altered DNA methylation patterns at imprinted genes. Int. J. Obes. (Lond.).

[33] Joubert BR (2016). DNA methylation in newborns and maternal smoking in pregnancy: Genome-wide consortium meta-analysis. Am. J. Hum. Genet..

[34] Soubry A (2016). Obesity-related DNA methylation at imprinted genes in human sperm: Results from the TIEGER study. Clin. Epigenet..

[35] Court F, Arnaud P (2017). An annotated list of bivalent chromatin regions in human ES cells: A new tool for cancer epigenetic research. Oncotarget.

[36] Rubino T (2009). Changes in hippocampal morphology and neuroplasticity induced by adolescent THC treatment are associated with cognitive impairment in adulthood. Hippocampus.

[37] Park HR (2016). A short review on the current understanding of autism spectrum disorders. Exp. Neurobiol..

[38] Sato D (2012). SHANK1 deletions in males with autism spectrum disorder. Am. J. Hum. Genet..

[39] Jiang YH, Ehlers MD (2013). Modeling autism by *SHANK* gene mutations in mice. Neuron.

[40] Grayson DR, Guidotti A (2016). Merging data from genetic and epigenetic approaches to better understand autistic spectrum disorder. Epigenomics.

[41] Vogel Ciernia A, La Salle J (2016). The landscape of DNA methylation amid a perfect storm of autism aetiologies. Nat. Rev. Neurosci..

[42] Gregory SG (2009). Genomic and epigenetic evidence for oxytocin receptor deficiency in autism. BMC Med..

[43] Lips EH (2013). Triple-negative breast cancer: BRCAness and concordance of clinical features with BRCA1-mutation carriers. Br. J. Cancer.

[44] Bernards SS (2018). Clinical characteristics and outcomes of patients with BRCA1 or RAD51C methylated versus mutated ovarian carcinoma. Gynecol. Oncol..

[45] Vos S, van Diest PJ, Moelans CB (2018). A systematic review on the frequency of *BRCA* promoter methylation in breast and ovarian carcinomas of *BRCA* germline mutation carriers: Mutually exclusive, or not?. Crit. Rev. Oncol. Hematol..

[46] Murphy SK (2012). Gender-specific methylation differences in relation to prenatal exposure to cigarette smoke. Gene.

[47] Dosemeci A, Weinberg RJ, Reese TS, Tao-Cheng JH (2016). The postsynaptic density: There is more than meets the eye. Front. Synaptic Neurosci..

[48] Xing J (2016). Resequencing and association analysis of six PSD-95-related genes as possible susceptibility genes for schizophrenia and autism spectrum disorders. Sci. Rep..

[49] Kaizuka T, Takumi T (2018). Postsynaptic density proteins and their involvement in neurodevelopmental disorders. J. Biochem..

[50] Rasmussen AH, Rasmussen HB, Silahtaroglu A (2017). The DLGAP family: Neuronal expression, function and role in brain disorders. Mol. Brain.

[51] Watson CT (2015). Genome-wide DNA methylation profiling reveals epigenetic changes in the rat nucleus accumbens associated with cross-generational effects of adolescent THC exposure. Neuropsychopharmacology.

[52] Bernstein BE (2006). A bivalent chromatin structure marks key developmental genes in embryonic stem cells. Cell.

[53] Thalheim T, Herberg M, Loeffler M, Galle J (2017). The regulatory capacity of bivalent genes—a theoretical approach. Int. J. Mol. Sci..

[54] Rakyan VK (2010). Human aging-associated DNA hypermethylation occurs preferentially at bivalent chromatin domains. Genome Res..

[55] Ohm JE (2007). A stem cell-like chromatin pattern may predispose tumor suppressor genes to DNA hypermethylation and heritable silencing. Nat. Genet..

[56] Corley MJ (2019). Epigenetic delay in the neurodevelopmental trajectory of DNA methylation states in autism spectrum disorders. Front. Genet..

[57] Hammoud SS (2009). Distinctive chromatin in human sperm packages genes for embryo development. Nature.

[58] Murphy KE, Jenkins TG, Carrell DT (2016). The Epigenome and Developmental Origins of Health and Disease, Ch. 18.

[59] Wu SF, Zhang H, Cairns BR (2011). Genes for embryo development are packaged in blocks of multivalent chromatin in zebrafish sperm. Genome Res..

[60] Hasin DS (2018). US epidemiology of cannabis use and associated problems. Neuropsychopharmacology.

[61] Payne KS, Mazur DJ, Hotaling JM, Pastuszak AW (2019). Cannabis and male fertility: A systematic review. J. Urol..

[62] Nassan FL (2019). Marijuana smoking and markers of testicular function among men from a fertility centre. Hum. Reprod..

[63] Jenkins TG (2017). Cigarette smoking significantly alters sperm DNA methylation patterns. Andrology.

[64] Morkve Knudsen GT (2019). Epigenome-wide association of father’s smoking with offspring DNA methylation: A hypothesis-generating study. Environ. Epigenet..

[65] Biederman J (2017). Is paternal smoking at conception a risk for ADHD? A controlled study in youth with and without ADHD. J. Atten. Disord..

[66] Bonnie RJ, Stratton K, Wallace RB (2007). Ending the Tobacco Problem: A Blueprint for the Nation.

[67] Hawkey AB (2019). Paternal nicotine exposure in rats produces long-lasting neurobehavioral effects in the offspring. Neurotoxicol. Teratol..

[68] Levin ED (2019). Paternal THC exposure in rats causes long-lasting neurobehavioral effects in the offspring. Neurotoxicol. Teratol..

[69] Bassil CF, Huang Z, Murphy SK (2013). Bisulfite pyrosequencing. Methods Mol. Biol..

[70] Shen, L. *GeneOverlap: Test and visualize gene overlaps. R package 1.20.0*. https://shenlab-sinai.github.io/shenlab-sinai/ (2019).

