# Supplementary material for: Sperm DNA methylation altered by THC and nicotine: Vulnerability of neurodevelopmental genes with bivalent chromatin
Source: Sci Rep. 2020 Sep 29;10:16022. doi: 10.1038/s41598-020-72783-0 (PMC7525661; doi:10.1038/s41598-020-72783-0)
Supplement: Supplementary file 1 — Supplementary Tables. [file 41598_2020_72783_MOESM1_ESM.pdf]

Sperm DNA Methylation Altered by THC and Nicotine: Vulnerability of Neurodevelopmental Genes with Bivalent Chromatin

Supplementary Materials

Rose Schrott[1,2], Maya Rajavel[1], Kelly Acharya[3], Zhiqing Huang[3], Chaitanya Acharya[4], Andrew Hawkey[5], Erica Pippen[5], H. Kim Lyerly[4], Edward D. Levin[5], *Susan K. Murphy[1,2,6].

[1]Department of Obstetrics and Gynecology, Division of Reproductive Sciences, Duke University Medical Center, Durham, North Carolina
[2] Integrated Toxicology and Environmental Health Program, Nicholas School of the Environment, Duke University, Durham, North Carolina
[3] Department of Obstetrics and Gynecology, Division of Reproductive Endocrinology and Infertility, Duke University Medical Center, Durham, North Carolina
[4] Center for Applied Therapeutics, Division of Surgical Sciences, Dept. of Surgery, Duke University Medical Center, Durham, North Carolina
[5] Department of Psychiatry and Behavioral Sciences, Duke University Medical Center, Durham, North Carolina
[6] Department of Pathology, Duke University Medical Center, Durham, North Carolina

Table S1: PCR conditions:

Gene Name	Forward Primer (5'→3')	Reverse Primer (5'→3')	Sequencing Primer (5'→3')	PCR Cycling Conditions*
Shank1	GATATTTGTAGGGTGTGGAATTTGAG	BTN-AAACCCCAAACAAAACTCCTC	GGGTGTGGAATTTGAGA	94-63-72 x 55
Syt3	BTN-GAGGAAGAAGGGATTTGTTTAGTTT	CTCTCACCAATCATAATCTACCACT	ACCAATCATAATCTACCACT	94-64/61-72 x 5 at each annealing temp, then 94-58-72 x 55
Lrrtm4	BTN-TGGATTTTTTTTATAATAAATTTTAGAT	ACTCCAAATAAAACTCTTTCAACTT	CAAAAAACCAACAAATACAT	94-64/61-72 x 5 at each annealing temp, then 94-58-72 x 55
Nrxn1	BTN-AGGTAGAATTTTTGGTTTAGTTTAG	CTAATCAATCCCCACAAAACATA	CTAAAATTTCACAAACCCT	94-62/60-72 x 5 at each annealing temp, then 94-58-72 x 55
Nrxn3	ATGTTAATTTGGTTTTGAAGGATG	BTN-TACCTAAAATAAACCTTCCAAACTC	GGTTTTTTTGGTTATTA	94-62/60-72 x 5 at each annealing temp, then 94-58-72 x 55
Dlg4	GGTAATTGGGGAAGTTTTTAGGATA	BTN-TTTAAAACCCCTCACCCTATACAA	AGGATAATTTTAAAGTTGGT	94-63-72 x 55
Grid1	BTN-GTGTTGTTGGTTTGAAAAAGGATAT	CAATTCACTTACACACCCACTTAT	CACACCCACTTATCCC	94-64-72 x 55

*All reactions were performed with a heated lid at 99 degrees. Reactions began at 95 degrees for 15 minutes. Following 30 seconds at each cycling temperature, samples were heated at 72 degrees for 10 minutes and then cooled at 4 degrees.

Supplementary Table 2. Significant Biological Process GO terms for genes entered into the String database. Bolded GO terms are related to neuronal processes.

#term ID	term description	observed gene count	background gene count	false discovery rate	matching proteins in your network (labels)
GO:0032502	developmental process	137	2616	1.45E-11	Adam15,Adora2a,Adra2c,Aldh1a3,Arf6,Arhgef28,Arrb2,Atat1,Atp1a3,Axin2,Bcan,Bmp6,Brinp3,Bsn,Cabp1,Capn2,Capn3,Cbx7,Cd8a,Cdc20,Cdh22,Cebpe,Celsr2,Celsr3,Chad,Chst11,Cln8,Crip1,Dapk3,Dbn1,Dgkg,Diexf,Dlg4,Dnmt3a,Dnmt3l,Dpf1,Dpysl5,Drc1,Dyx1c1,Elavl2,Ezr,Fat3,Fbxl15,Fcrla,Ffar2,Fgf14,Foxg1,Fzd9,Gata2,Gdf6,Gfi1,Gpr157,Grm4,Hgf,Hnf1a,Igfals,Igsf9,Inppl1,Jak3,Kazn,Kcne2,Lfng,Limk2,Llgl1,Mag,Map2k1,Mapt,Matk,Mical2,Mmp24,Msi1,Myc,Nell2,Ninj1,Nolc1,Nos3,Nr3c1,Nrg2,Nrxn1,Ntrk2,Odf2,Pacsin1,Palm,Pax8,Pcsk4,Pcsk9,Phox2a,Pkm,Plec,Plod3,Pomk,Pou2f1,Ppp1cc,Ppp1r15a,Prrc2a,Psd,RGD1311558,Rab3a,Rasgrp1,Rbp4,Rere,Rita1,Rogdi,S1pr1,Scn5a,Sh3bp1,Sh3gl2,Shank1,Siah1,Slc25a4,Slc39a3,Sorbs2,Spred2,Src,Sstr2,Stim1,Stk24,Ston2,Synj2,Syt3,Tbx3,Tbx5,Tdrd7,Tgfb3,Tgfbr2,Tox2,Trpm2,Ucp3,Unc13a,Unc5a,Uncx,Usp7,Vapa,Vegfb,Vwf,Zbtb42,Zfp37
GO:0030154	cell differentiation	93	1561	5.85E-10	Adam15,Adora2a,Adra2c,Arf6,Arhgef28,Bmp6,Brinp3,Capn2,Capn3,Cd8a,Cdc20,Cebpe,Celsr2,Celsr3,Chst11,Cln8,Dapk3,Dbn1,Dgkg,Dlg4,Dnmt3a,Dnmt3l,Dpysl5,Dyx1c1,Elavl2,Ezr,Fcrla,Ffar2,Foxg1,Fzd9,Gata2,Gdf6,Gfi1,Gpr157,Grm4,Hgf,Igsf9,Inppl1,Jak3,Kazn,Lfng,Limk2,Llgl1,Mag,Map2k1,Mapt,Matk,Mmp24,Msi1,Myc,Nolc1,Nr3c1,Nrxn1,Ntrk2,Odf2,Pacsin1,Pax8,Pcsk4,Pcsk9,Plec,Plod3,Pomk,Ppp1cc,Prrc2a,Psd,RGD1311558,Rab3a,Rasgrp1,Rere,Rita1,Rogdi,S1pr1,Sh3bp1,Sh3gl2,Shank1,Siah1,Sorbs2,Src,Stim1,Stk24,Ston2,Syt3,Tbx3,Tbx5,Tdrd7,Tgfb3,Tgfbr2,Trpm2,Unc13a,Unc5a,Uncx,Vapa,Zfp37
GO:0048869	cellular developmental process	94	1592	5.85E-10	Adam15,Adora2a,Adra2c,Arf6,Arhgef28,Bmp6,Brinp3,Capn2,Capn3,Cd8a,Cdc20,Cebpe,Celsr2,Celsr3,Chst11,Cln8,Dapk3,Dbn1,Dgkg,Dlg4,Dnmt3a,Dnmt3l,Dpysl5,Dyx1c1,Elavl2,Ezr,Fcrla,Ffar2,Foxg1,Fzd9,Gata2,Gdf6,Gfi1,Gpr157,Grm4,Hgf,Igsf9,Inppl1,Jak3,Kazn,Lfng,Limk2,Llgl1,Mag,Map2k1,Mapt,Matk,Mmp24,Msi1,Myc,Nolc1,Nr3c1,Nrxn1,Ntrk2,Odf2,Pacsin1,Pax8,Pcsk4,Pcsk9,Plec,Plod3,Pomk,Ppp1cc,Prrc2a,Psd,RGD1311558,Rab3a,Rasgrp1,Rere,Rita1,Rogdi,S1pr1,Sh3bp1,Sh3gl2,Shank1,Siah1,Slc39a3,Sorbs2,Src,Stim1,Stk24,Ston2,Syt3,Tbx3,Tbx5,Tdrd7,Tgfb3,Tgfbr2,Trpm2,Unc13a,Unc5a,Uncx,Vapa,Zfp37
GO:0048856	anatomical structure development	122	2396	1.17E-09	Adam15,Adora2a,Adra2c,Aldh1a3,Arf6,Arrb2,Atat1,Atp1a3,Axin2,Bcan,Bmp6,Brinp3,Bsn,Cabp1,Capn2,Capn3,Cbx7,Cd8a,Cdc20,Cdh22,Cebpe,Celsr2,Celsr3,Chad,Chst11,Cln8,Crip1,Dapk3,Dbn1,Dgkg,Diexf,Dlg4,Dnmt3a,Dpf1,Dpysl5,Drc1,Dyx1c1,Elavl2,Ezr,Fat3,Fbxl15,Fgf14,Foxg1,Fzd9,Gata2,Gdf6,Gfi1,Gpr157,Grm4,Hgf,Hnf1a,Igsf9,Inppl1,Jak3,Kazn,Kcne2,Lfng,Limk2,Llgl1,Mag,Map2k1,Mapt,Mical2,Mmp24,Msi1,Myc,Ninj1,Nolc1,Nos3,Nr3c1,Nrg2,Nrxn1,Ntrk2,Odf2,Pacsin1,Palm,Pax8,Pcsk4,Pcsk9,Pkm,Plec,Plod3,Pomk,Pou2f1,Ppp1cc,Psd,RGD1311558,Rab3a,Rbp4,Rere,Rita1,Rogdi,S1pr1,Scn5a,Sh3bp1,Sh3gl2,Shank1,Siah1,Slc25a4,Slc39a3,Sorbs2,Spred2,Src,Sstr2,Stim1,Stk24,Ston2,Synj2,Tbx3,Tbx5,Tdrd7,Tgfb3,Tgfbr2,Tox2,Trpm2,Unc5a,Uncx,Usp7,Vapa,Vegfb,Vwf,Zbtb42
GO:0007275	multicellular organism development	116	2257	2.19E-09	Adam15,Adora2a,Adra2c,Aldh1a3,Arf6,Arrb2,Atat1,Atp1a3,Axin2,Bcan,Bmp6,Brinp3,Bsn,Cabp1,Capn2,Cbx7,Cd8a,Cdc20,Cdh22,Cebpe,Celsr2,Celsr3,Chad,Chst11,Cln8,Crip1,Dapk3,Dbn1,Dgkg,Diexf,Dlg4,Dnmt3a,Dpf1,Dpysl5,Drc1,Dyx1c1,Elavl2,Fat3,Fbxl15,Fgf14,Foxg1,Fzd9,Gata2,Gdf6,Gfi1,Gpr157,Grm4,Hgf,Hnf1a,Igsf9,Inppl1,Jak3,Kazn,Kcne2,Lfng,Limk2,Llgl1,Mag,Map2k1,Mapt,Mical2,Mmp24,Myc,Ninj1,Nolc1,Nos3,Nr3c1,Nrg2,Nrxn1,Ntrk2,Odf2,Pacsin1,Palm,Pax8,Pcsk9,Pkm,Plod3,Pomk,Pou2f1,Ppp1cc,Psd,RGD1311558,Rab3a,Rbp4,Rere,Rita1,Rogdi,S1pr1,Scn5a,Sh3gl2,Shank1,Siah1,Slc25a4,Slc39a3,Sorbs2,Spred2,Src,Sstr2,Stim1,Stk24,Ston2,Synj2,Tbx3,Tbx5,Tdrd7,Tgfb3,Tgfbr2,Tox2,Trpm2,Unc5a,Uncx,Usp7,Vapa,Vegfb,Vwf,Zbtb42
GO:0048731	system development	107	2034	4.15E-09	Adam15,Adora2a,Adra2c,Aldh1a3,Arf6,Arrb2,Atat1,Atp1a3,Bcan,Bmp6,Brinp3,Bsn,Cbx7,Cd8a,Cdc20,Cdh22,Cebpe,Celsr2,Celsr3,Chad,Chst11,Cln8,Crip1,Dapk3,Dbn1,Dgkg,Dlg4,Dnmt3a,Dpf1,Dpysl5,Drc1,Dyx1c1,Elavl2,Fbxl15,Fgf14,Foxg1,Fzd9,Gata2,Gdf6,Gfi1,Gpr157,Grm4,Hgf,Hnf1a,Igsf9,Inppl1,Jak3,Kazn,Kcne2,Lfng,Limk2,Llgl1,Mag,Map2k1,Mapt,Mical2,Mmp24,Myc,Ninj1,Nolc1,Nos3,Nr3c1,Nrg2,Nrxn1,Ntrk2,Pacsin1,Palm,Pax8,Pcsk9,Pkm,Plod3,Pomk,Pou2f1,Ppp1cc,Psd,RGD1311558,Rab3a,Rbp4,Rere,Rita1,Rogdi,S1pr1,Scn5a,Sh3gl2,Shank1,Slc25a4,Slc39a3,Sorbs2,Src,Sstr2,Stim1,Stk24,Ston2,Synj2,Tbx3,Tbx5,Tdrd7,Tgfb3,Tgfbr2,Tox2,Trpm2,Unc5a,Uncx,Vapa,Vegfb,Vwf,Zbtb42
GO:0032501	multicellular organismal process	137	2936	8.51E-09	Adam15,Adk,Adora2a,Adra2c,Aldh1a3,Arf6,Arrb2,Atat1,Atp1a3,Avp,Axin2,B4galnt1,Bcan,Bmp6,Brinp3,Bsn,Cabp1,Cacna1a,Cacna1g,Cacna1i,Capn2,Cbx7,Cd8a,Cdc20,Cdh22,Cebpe,Celsr2,Celsr3,Chad,Chst11,Cln8,Crip1,Dapk3,Dbn1,Dgkg,Diexf,Dlg4,Dnmt3a,Dnmt3l,Dpf1,Dpysl5,Drc1,Dyx1c1,Elavl2,Ezr,Fam129b,Fat3,Fbxl15,Fgf12,Fgf14,Foxg1,Fzd9,Gata2,Gdf6,Gfi1,Gjd2,Gpr157,Grid1,Grik3,Grm4,Hgf,Hnf1a,Igsf9,Inppl1,Jak3,Kazn,Kcne2,Kcnmb4,Lfng,Limk2,Llgl1,Mag,Map2k1,Mapt,Mical2,Mmp24,Myc,Ninj1,Nolc1,Nos3,Nr3c1,Nrg2,Nrxn1,Ntrk2,Odf2,Pacsin1,Palm,Pax8,Pcsk4,Pcsk9,Pgc,Pkm,Plec,Plod3,Pomk,Pou2f1,Ppp1cc,Prkaca,Psd,RGD1311558,Rab3a,Rasgrp1,Rbp4,Rere,Rita1,Rogdi,S1pr1,Scamp4,Scn5a,Sh3gl2,Shank1,Siah1,Slc25a4,Slc39a3,Sorbs2,Spred2,Src,Sstr2,Stim1,Stk24,Ston2,Synj2,Tbx3,Tbx5,Tdrd7,Tgfb3,Tgfbr2,Tox2,Trim63,Trpm2,Unc5a,Uncx,Usp7,Vapa,Vegfb,Vwf,Zbtb42
GO:0050789	regulation of biological process	184	4461	2.32E-08	Acsl5,Adam15,Adk,Adora2a,Adra2c,Ank3,Anp32b,Arf6,Arhgap27,Arhgef28,Arpc1a,Arrb2,Atat1,Atp1a3,Avp,Axin2,Bcan,Bmp6,Bop1,Brinp3,C4a,Cabp1,Cacna1a,Cacna1g,Cacna1i,Cacna2d1,Cacna2d2,Capn2,Capn3,Cbx7,Ccdc80,Cd8a,Cdc20,Cebpe,Celsr2,Celsr3,Chad,Chd6,Chst11,Cln8,Crip1,Csdc2,Csnk1g2,Dapk3,Dbn1,Dgkg,Dkc1,Dlg4,Dnmt3a,Dnmt3l,Dok3,Dpf1,Dyrk3,Dyx1c1,ENSRNOG00000050782,Elavl2,Ezr,Fam129b,Fbxl15,Ffar2,Fgf12,Fgf14,Fgfr4,Fkbpl,Foxg1,Fto,Fzd9,Galr3,Gata2,Gdf6,Gfi1,Gnb2,Gng7,Gpr157,Gpr6,Grasp,Grb7,Grid1,Grik3,Grina,Grm4,Hgf,Hipk4,Hnf1a,Igfbp6,Igsf9,Inppl1,Itpr3,Jak3,Kcne2,Kcng1,Kcnip1,Kcnip4,Kcnj4,Kdm8,Lfng,Limk2,Llgl1,Lrrtm4,Lzts2,Mag,Map2k1,Mapt,Masp1,Matk,Mbnl2,Mefv,Mical2,Myc,Nell2,Nfe2,Ninj1,Nolc1,Nos3,Nr3c1,Nrg2,Nrxn1,Ntrk2,Oasl,Pacsin1,Palm,Pax8,Pcsk9,Pgc,Phox2a,Pif1,Pla2g1b,Pomt1,Por,Pou2f1,Ppfia3,Ppp1cc,Ppp1r14a,Ppp1r15a,Prkaca,Psd,RGD1311558,Rab3a,Rasgrp1,Rassf9,Rbp4,Rere,Rita1,Rnf187,Rogdi,S1pr1,Sbno1,Scn5a,Sh3bp1,Sh3gl2,Sh3rf2,Shank1,Siah1,Slc15a1,Slc25a4,Spred2,Src,Sstr2,Stim1,Stk24,Ston2,Stpg1,Syt3,Taf6,Tbx3,Tbx5,Tdrd7,Tes,Tgfb3,Tgfbr2,Tle3,Tox2,Trim63,Trpm2,Tsks,Unc13a,Unc5a,Uncx,Usp7,Vegfb,Zbtb42,Zbtb8a,Zfp37,Zfp90

GO:0065007	biological regulation	192	4801	8.41E-08	Acsl5,Adam15,Adk,Adora2a,Adra2c,Aldh1a3,Ank3,Anp32b,Arf6,Arhgap27,Arhgef28,Arpc1a,Arrb2,Atat1,Atp1a3,Avp,Axin2,B4galnt1,Bcan,Bmp6,Bop1,Brinp3,C4a,Cabp1,Cacna1a,Cacna1g,Cacna1i,Cacna2d1,Cacna2d2,Capn2,Capn3,Cbx7,Ccdc80,Cd8a,Cdc20,Cebpe,Celsr2,Celsr3,Chad,Chd6,Chst11,Cln8,Crip1,Csdc2,Csnk1g2,Dapk3,Dbn1,Dgkg,Dkc1,Dlg4,Dnmt3a,Dnmt3l,Dok3,Dpf1,Dyrk3,Dyx1c1,ENSRNOG00000050782,Elavl2,Ezr,Fam129b,Fbxl15,Ffar2,Fgf12,Fgf14,Fgfr4,Fkbpl,Foxg1,Fto,Fzd9,Galr3,Gata2,Gdf6,Gfi1,Gjd2,Gnb2,Gng7,Gpr157,Gpr6,Grasp,Grb7,Grid1,Grik3,Grina,Grm4,Hgf,Hipk4,Hnf1a,Igfbp6,Igsf9,Inppl1,Itpr3,Jak3,Kcne2,Kcng1,Kcnip1,Kcnip4,Kcnj4,Kcnk1,Kcnmb4,Kdm8,Lfng,Limk2,Llgl1,Lrrtm4,Lzts2,Mag,Map2k1,Mapt,Masp1,Matk,Mbnl2,Mefv,Mical2,Myc,Nell2,Nfe2,Ninj1,Nolc1,Nos3,Nr3c1,Nrg2,Nrxn1,Ntrk2,Oasl,Pacsin1,Palm,Pax8,Pcsk9,Pgc,Phox2a,Pif1,Pla2g1b,Plod3,Pomt1,Por,Pou2f1,Ppfia3,Ppp1cc,Ppp1r14a,Ppp1r15a,Prkaca,Psd,RGD1311558,Rab3a,Rasgrp1,Rassf9,Rbp4,Rere,Rita1,Rnf187,Rogdi,S1pr1,Sbno1,Scn5a,Sh3bp1,Sh3gl2,Sh3rf2,Shank1,Siah1,Slc15a1,Slc25a4,Slc39a3,Spred2,Src,Sstr2,Stim1,Stk24,Ston2,Stpg1,Syt3,Taf6,Tbx3,Tbx5,Tdrd7,Tes,Tgfb3,Tgfbr2,Tle3,Tox2,Trim63,Trpm2,Tsks,Unc13a,Unc5a,Uncx,Usp7,Vegfb,Vwf,Zbtb42,Zbtb8a,Zfp37,Zfp90
**GO:0007399**	**nervous system development**	**71**	**1219**	**2.50E-07**	**Adora2a,Adra2c,Aldh1a3,Arf6,Arrb2,Atat1,Atp1a3,Bcan,Bmp6,Brinp3,Bsn,Cdc20,Cdh22,Celsr2,Celsr3,Cln8,Dapk3,Dbn1,Dgkg,Dlg4,Dnmt3a,Dpf1,Dpysl5,Dyx1c1,Elavl2,Fgf14,Foxg1,Fzd9,Gata2,Gdf6,Gfi1,Gpr157,Grm4,Hgf,Igsf9,Inppl1,Llgl1,Mag,Map2k1,Mapt,Mmp24,Myc,Nr3c1,Nrxn1,Ntrk2,Pacsin1,Palm,Pax8,Pcsk9,Plod3,Pomk,Ppp1cc,Psd,RGD1311558,Rab3a,Rere,Rita1,Rogdi,S1pr1,Scn5a,Sh3gl2,Shank1,Src,Sstr2,Stk24,Synj2,Tbx3,Tgfbr2,Unc5a,Uncx,Vapa**
GO:0009987	cellular process	226	6022	2.50E-07	Abcd2,Acsf2,Acsl5,Actn1,Adam15,Adk,Adora2a,Adra2c,Aldh1a3,Ank3,Anp32b,Arf6,Arhgap27,Arhgef28,Arpc1a,Arrb2,Atat1,Atp1a3,Avp,Axin2,B4galnt1,B4galt4,Bet1,Bmp6,Bop1,Brinp3,Bsn,Cacna1a,Cacna1g,Cacna1i,Cacna2d1,Capn2,Capn3,Cbx7,Ccdc80,Cd8a,Cdc20,Cebpe,Celsr2,Celsr3,Chad,Chd6,Chst11,Cln8,Clvs2,Crip1,Csdc2,Csnk1g2,Cyb5a,Dapk3,Dbn1,Dgkg,Diexf,Dkc1,Dlg4,Dnmt3a,Dnmt3l,Dok3,Dpf1,Dpysl5,Drc1,Dyrk3,Dyx1c1,ENSRNOG00000050782,Elavl2,Ezr,Fam129b,Fbxl15,Fcrla,Ffar2,Fgf12,Fgfr4,Foxg1,Fto,Fzd9,Galr3,Gata2,Gdf6,Gfi1,Gjd2,Gnb2,Gng7,Gpr157,Gpr6,Grasp,Grb7,Grid1,Grik3,Grina,Grm4,Gypc,Hal,Hgf,Hipk4,Hnf1a,Igfals,Igsf9,Inppl1,Itpr3,Jak3,Kazn,Kcne2,Kcng1,Kcnip1,Kcnip4,Kcnj4,Kcnk1,Kcnmb4,Kdm8,Lfng,Limk2,Llgl1,Lrrtm4,Lzts2,Mag,Map2k1,Mapre2,Mapt,Matk,Mbnl2,Mical2,Mmp24,Msi1,Msrb2,Mtr,Myc,Nell2,Nfe2,Nmnat2,Nolc1,Nos3,Nr3c1,Nrg2,Nrxn1,Ntrk2,Odf2,Oplah,Pacsin1,Palm,Pax8,Pcsk4,Pcsk9,Pcyt2,Pgc,Phox2a,Pif1,Pip5k1c,Pkm,Pla2g1b,Plec,Plod1,Plod3,Pomk,Pomt1,Por,Pou2f1,Ppfia3,Ppp1cc,Ppp1r14a,Ppp1r15a,Prkaca,Prrc2a,Psd,Qtrt1,RGD1311558,RGD1559696,Rab31,Rab3a,Rasgrp1,Rassf9,Rbp4,Repin1,Rere,Rita1,Rnf182,Rnf187,Rnf44,Rogdi,Rpl35a,Rtn3,S1pr1,Scn5a,Sh3bp1,Sh3gl2,Sh3rf2,Shank1,Siah1,Slc25a4,Slc39a3,Sorbs2,Src,Sstr2,Stim1,Stk24,Ston2,Stpg1,Synj2,Syt3,Taf6,Tbx3,Tbx5,Tdrd7,Tgfb3,Tgfbr2,Tle3,Tmem14c,Tox2,Tppp3,Trim63,Trmt11,Trpm2,Tubb5,U2af1l4,Ucp3,Unc13a,Unc5a,Uncx,Usp7,Vapa,Vegfb,Vwf,Zbtb42,Zbtb8a,Zdhhc22,Zfp37,Zfp90
GO:0050794	regulation of cellular process	169	4191	7.73E-07	Acsl5,Adam15,Adk,Adora2a,Adra2c,Ank3,Anp32b,Arf6,Arhgap27,Arhgef28,Arpc1a,Arrb2,Atat1,Atp1a3,Avp,Axin2,Bcan,Bmp6,Bop1,Brinp3,C4a,Cabp1,Cacna1g,Cacna1i,Capn2,Capn3,Cbx7,Cd8a,Cdc20,Cebpe,Celsr2,Celsr3,Chad,Chd6,Chst11,Cln8,Crip1,Csdc2,Csnk1g2,Dapk3,Dbn1,Dgkg,Dkc1,Dlg4,Dnmt3a,Dnmt3l,Dok3,Dpf1,Dyrk3,Dyx1c1,ENSRNOG00000050782,Elavl2,Ezr,Fam129b,Fbxl15,Ffar2,Fgf12,Fgf14,Fgfr4,Foxg1,Fzd9,Galr3,Gata2,Gdf6,Gfi1,Gnb2,Gng7,Gpr157,Gpr6,Grasp,Grb7,Grid1,Grik3,Grina,Grm4,Hgf,Hipk4,Hnf1a,Igfbp6,Igsf9,Inppl1,Itpr3,Jak3,Kcne2,Kcng1,Kcnip1,Kcnip4,Kdm8,Lfng,Limk2,Llgl1,Lrrtm4,Lzts2,Mag,Map2k1,Mapt,Matk,Mbnl2,Mefv,Mical2,Myc,Nell2,Nfe2,Nolc1,Nos3,Nr3c1,Nrg2,Nrxn1,Ntrk2,Pacsin1,Palm,Pax8,Pcsk9,Phox2a,Pif1,Pla2g1b,Pomt1,Por,Pou2f1,Ppfia3,Ppp1cc,Ppp1r14a,Ppp1r15a,Prkaca,Psd,RGD1311558,Rab3a,Rasgrp1,Rassf9,Rbp4,Rere,Rita1,Rnf187,Rogdi,S1pr1,Sbno1,Scn5a,Sh3bp1,Sh3gl2,Sh3rf2,Shank1,Siah1,Slc25a4,Spred2,Src,Sstr2,Stk24,Ston2,Stpg1,Syt3,Taf6,Tbx3,Tbx5,Tes,Tgfb3,Tgfbr2,Tle3,Tox2,Trpm2,Tsks,Unc13a,Unc5a,Uncx,Usp7,Vegfb,Zbtb42,Zbtb8a,Zfp37,Zfp90
GO:0009653	anatomical structure morphogenesis	48	734	5.55E-06	Adam15,Adora2a,Bmp6,Capn2,Capn3,Celsr2,Celsr3,Chad,Chst11,Crip1,Dlg4,Dpysl5,Foxg1,Gata2,Hgf,Hnf1a,Inppl1,Lfng,Llgl1,Map2k1,Mical2,Myc,Ninj1,Nolc1,Nrxn1,Pacsin1,Pax8,Plod3,RGD1311558,Rab3a,Rere,Rogdi,S1pr1,Scn5a,Sh3bp1,Sh3gl2,Shank1,Slc39a3,Src,Stim1,Stk24,Tbx3,Tbx5,Tdrd7,Tgfb3,Tgfbr2,Unc5a,Vegfb
**GO:0022008**	**neurogenesis**	**51**	**839**	**1.66E-05**	**Adora2a,Adra2c,Arf6,Bmp6,Brinp3,Cdc20,Celsr2,Celsr3,Cln8,Dapk3,Dbn1,Dgkg,Dlg4,Dnmt3a,Dpysl5,Dyx1c1,Elavl2,Fzd9,Gata2,Gdf6,Gfi1,Gpr157,Grm4,Hgf,Igsf9,Inppl1,Llgl1,Mag,Map2k1,Mapt,Mmp24,Myc,Nr3c1,Nrxn1,Ntrk2,Pacsin1,Pcsk9,Pomk,Ppp1cc,Psd,RGD1311558,Rab3a,Rere,Rita1,Rogdi,S1pr1,Sh3gl2,Shank1,Stk24,Unc5a,Vapa**
GO:0051179	localization	101	2237	1.74E-05	Abcd2,Acsl5,Ank3,Anks1b,Anp32b,Anxa6,Arf6,Arhgap27,Arrb2,Asic4,Atp1a3,B4galnt1,Bet1,Bsn,Cacna1a,Cacna1g,Cacna1i,Cacna2d1,Cacna2d2,Capn3,Cebpe,Celsr3,Chst11,Cln8,Csnk1g2,Dlg4,Drc1,Dyx1c1,Ezr,Fam129b,Ffar2,Fgfr4,Gata2,Grasp,Grid1,Grik3,Grina,Grm4,Hare,Hgf,Hnf1a,Itpr3,Jak3,Kcne2,Kcng1,Kcnip1,Kcnip4,Kcnj4,Kcnk1,Kcnmb4,Klc1,Llgl1,Map2k1,Myc,Nrxn1,Ntrk2,Odf2,Pacsin1,Palm,Pcsk9,Pip5k1c,Pla2g1b,Plod3,Pomk,Ppfia3,Psd,RGD1311558,RGD1559696,Rab31,Rab3a,Rasgrp1,Rassf9,Rbp4,Rere,Rita1,Rtn3,S1pr1,Scamp4,Scn5a,Sh3bp1,Sh3gl2,Shank1,Slc10a4,Slc15a1,Slc25a4,Slc39a3,Slc3a2,Src,Stim1,Ston2,Stpg1,Svop,Synj2,Syt3,Tbx3,Tgfbr2,Trpm2,Ucp3,Unc13a,Vapa,Zdhhc22
GO:0048518	positive regulation of biological process	107	2425	1.88E-05	Acsl5,Adk,Adora2a,Adra2c,Ank3,Anp32b,Arf6,Arpc1a,Arrb2,Atat1,Avp,Axin2,Bmp6,Brinp3,C4a,Cacna1g,Cacna1i,Cacna2d1,Capn3,Cbx7,Ccdc80,Cdc20,Cebpe,Chd6,Dapk3,Dbn1,Dkc1,Dlg4,Dnmt3a,Dnmt3l,Elavl2,Ezr,Fam129b,Fbxl15,Ffar2,Fgf12,Fgf14,Fgfr4,Fzd9,Galr3,Gata2,Gdf6,Gfi1,Grb7,Hgf,Hnf1a,Jak3,Kcne2,Kcnip1,Kdm8,Lfng,Lzts2,Mag,Map2k1,Mapt,Masp1,Mefv,Mical2,Myc,Nell2,Nfe2,Ninj1,Nolc1,Nos3,Nr3c1,Nrxn1,Ntrk2,Pacsin1,Palm,Pax8,Pcsk9,Pgc,Phox2a,Pla2g1b,Pomt1,Por,Ppp1cc,Ppp1r15a,Prkaca,RGD1311558,Rab3a,Rasgrp1,Rbp4,Rere,Rnf187,Rogdi,S1pr1,Scn5a,Sh3gl2,Shank1,Siah1,Slc25a4,Src,Stim1,Stk24,Stpg1,Syt3,Tbx3,Tbx5,Tgfb3,Tgfbr2,Tox2,Trpm2,Unc13a,Usp7,Vegfb,Zfp90
**GO:0048699**	**generation of neurons**	**47**	**781**	**6.07E-05**	**Adora2a,Adra2c,Arf6,Bmp6,Brinp3,Cdc20,Celsr2,Celsr3,Cln8,Dapk3,Dbn1,Dgkg,Dlg4,Dnmt3a,Dpysl5,Dyx1c1,Elavl2,Fzd9,Gata2,Gdf6,Gfi1,Grm4,Hgf,Igsf9,Inppl1,Llgl1,Mag,Map2k1,Mapt,Myc,Nr3c1,Nrxn1,Ntrk2,Pacsin1,Pcsk9,Pomk,Ppp1cc,Psd,RGD1311558,Rab3a,Rere,S1pr1,Sh3gl2,Shank1,Stk24,Unc5a,Vapa**
GO:0050896	response to stimulus	142	3604	6.44E-05	Acsl5,Adam15,Adora2a,Adra2c,Aldh1a3,Ank3,Arhgap27,Arhgef28,Arrb2,Atp1a3,Avp,Axin2,Bmp6,Brinp3,C4a,Cabp1,Cacna1g,Capn2,Capn3,Car5b,Cbx7,Ccdc80,Cd8a,Cebpe,Celsr2,Celsr3,Chad,Chd6,Cln8,Crip1,Csnk1g2,Cyb5a,Dapk3,Dbn1,Dgkg,Dnmt3a,Dnmt3l,Dok3,Dpysl5,ENSRNOG00000050782,Elavl2,Ezr,Ffar2,Fgf12,Fgfr4,Fto,Fzd9,Galr3,Gata2,Gdf6,Gfi1,Gnb2,Gng7,Gpr157,Gpr6,Grasp,Grb7,Grid1,Grik3,Grm4,Hare,Hgf,Hnf1a,Igfals,Inppl1,Itpr3,Jak3,Kcne2,Kcnj4,Kcnk1,Kcnmb4,Lfng,Limk2,Lrrtm4,Lzts2,Mag,Map2k1,Mapt,Masp1,Matk,Mefv,Mmp24,Msi1,Msrb2,Mtr,Myc,Ninj1,Nlrx1,Nolc1,Nos3,Nr3c1,Nrg2,Nrxn1,Ntrk2,Oasl,Palm,Pax8,Pcsk9,Pif1,Pip5k1c,Pkm,Pla2g1b,Plec,Plod1,Plod3,Pomt1,Por,Ppp1cc,Ppp1r14a,Ppp1r15a,Prkaca,RGD1311558,Rab31,Rab3a,Rasgrp1,Rassf9,Rbp4,Rita1,S1pr1,Scn5a,Sh3bp1,Sh3gl2,Shank1,Slc25a4,Src,Sstr2,Stim1,Stk24,Syt3,Tbx3,Tgfb3,Tgfbr2,Tle3,Tox2,Trim63,Trpm2,Ucp3,Unc13a,Unc5a,Usp7,Vegfb,Vwf

GO:0048522	positive regulation of cellular process	97	2201	7.71E-05	Acsl5,Adk,Adora2a,Adra2c,Ank3,Anp32b,Arf6,Arpc1a,Arrb2,Atat1,Avp,Axin2,Bmp6,Brinp3,Cacna1g,Cacna1i,Capn3,Cbx7,Cdc20,Cebpe,Chd6,Dapk3,Dbn1,Dkc1,Dlg4,Dnmt3a,Dnmt3l,Elavl2,Ezr,Fam129b,Fbxl15,Ffar2,Fgfr4,Fzd9,Galr3,Gata2,Gdf6,Gfi1,Grb7,Hgf,Hnf1a,Jak3,Kcne2,Kdm8,Lfng,Lzts2,Mag,Map2k1,Mapt,Mefv,Mical2,Myc,Nell2,Nfe2,Nolc1,Nos3,Nr3c1,Nrxn1,Ntrk2,Pacsin1,Palm,Pax8,Pcsk9,Phox2a,Pla2g1b,Pomt1,Por,Ppp1cc,Ppp1r15a,Prkaca,RGD1311558,Rab3a,Rasgrp1,Rbp4,Rere,Rnf187,Rogdi,S1pr1,Scn5a,Sh3gl2,Shank1,Siah1,Slc25a4,Src,Stk24,Stpg1,Syt3,Tbx3,Tbx5,Tgfb3,Tgfbr2,Tox2,Trpm2,Unc13a,Usp7,Vegfb,Zfp90
GO:0032879	regulation of localization	68	1378	0.00013	Acsl5,Adam15,Adora2a,Ank3,Arf6,Arrb2,Avp,Bmp6,Cabp1,Cacna1a,Cacna1g,Cacna1i,Cacna2d1,Cacna2d2,Capn3,Celsr3,Dapk3,Dbn1,Dlg4,Ezr,Ffar2,Fgf12,Fgf14,Fzd9,Gata2,Grb7,Hgf,Hnf1a,Itpr3,Jak3,Kcne2,Kcng1,Kcnip1,Kcnip4,Kcnj4,Llgl1,Map2k1,Myc,Nell2,Nolc1,Nr3c1,Nrxn1,Ntrk2,Pacsin1,Pax8,Pcsk9,Pla2g1b,Ppp1cc,RGD1311558,Rab3a,Rasgrp1,Rbp4,S1pr1,Scn5a,Sh3gl2,Shank1,Slc15a1,Slc25a4,Src,Stim1,Stk24,Ston2,Syt3,Tbx5,Tgfb3,Tgfbr2,Trpm2,Vegfb
GO:0065009	regulation of molecular function	71	1462	0.00013	Adam15,Adora2a,Adra2c,Ank3,Anp32b,Arhgap27,Arhgef28,Arrb2,Avp,Axin2,Bmp6,C4a,Cabp1,Cacna1a,Cacna2d1,Cacna2d2,Capn3,Cdc20,Chad,Dbn1,Dlg4,Dnmt3l,Fam129b,Fgf12,Fgf14,Fgfr4,Galr3,Gdf6,Gfi1,Gnb2,Hgf,Inppl1,Kcne2,Kcng1,Kcnip1,Kcnip4,Kcnmb4,Lfng,Llgl1,Lrrtm4,Map2k1,Myc,Nos3,Nrg2,Nrxn1,Ntrk2,Palm,Pcsk9,Pif1,Por,Ppp1r14a,Psd,Rab3a,Rasgrp1,S1pr1,Sh3bp1,Sh3rf2,Shank1,Slc15a1,Slc25a4,Src,Stim1,Stk24,Stpg1,Taf6,Tgfb3,Tgfbr2,Tsks,Usp7,Vegfb,Zfp90
GO:0048513	animal organ development	69	1420	0.00017	Adam15,Aldh1a3,Arf6,Arrb2,Atat1,Atp1a3,Bmp6,Cbx7,Cd8a,Cdh22,Cebpe,Chad,Chst11,Cln8,Crip1,Dbn1,Drc1,Fbxl15,Foxg1,Fzd9,Gata2,Gfi1,Hgf,Hnf1a,Inppl1,Jak3,Kazn,Kcne2,Lfng,Limk2,Mapt,Mical2,Myc,Ninj1,Nolc1,Nos3,Nr3c1,Nrg2,Nrxn1,Ntrk2,Pax8,Pcsk9,Pkm,Plod3,Pomk,Pou2f1,Rab3a,Rbp4,Rere,Rogdi,S1pr1,Scn5a,Slc25a4,Slc39a3,Sorbs2,Src,Sstr2,Stim1,Ston2,Synj2,Tbx3,Tbx5,Tdrd7,Tgfb3,Tgfbr2,Tox2,Trpm2,Vwf,Zbtb42
GO:0010646	regulation of cell communication	70	1486	0.00039	Adora2a,Adra2c,Ank3,Arf6,Arhgef28,Arrb2,Avp,Axin2,Bcan,Bmp6,Bop1,Cabp1,Capn3,Cdc20,Chad,Chst11,Dapk3,Dbn1,Dlg4,Dyrk3,Dyx1c1,Ezr,Fbxl15,Ffar2,Fgf12,Fgf14,Fgfr4,Fzd9,Gata2,Gdf6,Gfi1,Grb7,Grik3,Grina,Grm4,Hgf,Hipk4,Hnf1a,Igfbp6,Inppl1,Lfng,Lrrtm4,Map2k1,Mapt,Nell2,Nr3c1,Nrg2,Nrxn1,Ntrk2,Palm,Pax8,Pcsk9,Por,Ppfia3,Prkaca,Psd,Rab3a,Rasgrp1,Rbp4,Rita1,Sh3bp1,Shank1,Siah1,Spred2,Src,Stk24,Tgfb3,Trpm2,Unc13a,Vegfb
GO:0051049	regulation of transport	54	1043	0.00046	Acsl5,Adora2a,Ank3,Arrb2,Avp,Bmp6,Cabp1,Cacna1a,Cacna1g,Cacna1i,Cacna2d1,Cacna2d2,Capn3,Dbn1,Dlg4,Ezr,Ffar2,Fgf12,Fgf14,Gata2,Hnf1a,Jak3,Kcne2,Kcng1,Kcnip1,Kcnip4,Kcnj4,Llgl1,Map2k1,Myc,Nell2,Nolc1,Nr3c1,Nrxn1,Ntrk2,Pacsin1,Pax8,Pcsk9,Pla2g1b,Ppp1cc,Rab3a,Rasgrp1,Rbp4,Scn5a,Sh3gl2,Shank1,Slc15a1,Slc25a4,Src,Stim1,Ston2,Syt3,Tgfb3,Trpm2
**GO:0030182**	**neuron differentiation**	**31**	**460**	**0.00057**	**Adora2a,Celsr2,Celsr3,Cln8,Dapk3,Dgkg,Dlg4,Dnmt3a,Dpysl5,Elavl2,Gata2,Grm4,Igsf9,Llgl1,Map2k1,Mapt,Nrxn1,Ntrk2,Pacsin1,Pcsk9,Ppp1cc,Psd,RGD1311558,Rab3a,Rere,S1pr1,Sh3gl2,Shank1,Stk24,Unc5a,Vapa**
GO:0023051	regulation of signaling	69	1494	0.00077	Adora2a,Adra2c,Arf6,Arhgef28,Arrb2,Avp,Axin2,Bcan,Bmp6,Bop1,Cabp1,Capn3,Cdc20,Chad,Chst11,Dapk3,Dbn1,Dlg4,Dyrk3,Dyx1c1,Ezr,Fbxl15,Ffar2,Fgf12,Fgf14,Fgfr4,Fzd9,Gata2,Gdf6,Gfi1,Grb7,Grik3,Grina,Grm4,Hgf,Hipk4,Hnf1a,Igfbp6,Inppl1,Lfng,Lrrtm4,Map2k1,Mapt,Nell2,Nr3c1,Nrg2,Nrxn1,Ntrk2,Palm,Pax8,Pcsk9,Por,Ppfia3,Prkaca,Psd,Rab3a,Rasgrp1,Rbp4,Rita1,Sh3bp1,Shank1,Siah1,Spred2,Src,Stk24,Tgfb3,Trpm2,Unc13a,Vegfb
GO:0065008	regulation of biological quality	82	1883	0.00077	Adora2a,Adra2c,Aldh1a3,Ank3,Arf6,Arpc1a,Atp1a3,Avp,B4galnt1,Bcan,Bmp6,Cabp1,Cacna1a,Cacna1g,Cacna1i,Cacna2d1,Capn3,Cdc20,Cln8,Csdc2,Csnk1g2,Dapk3,Dbn1,Dlg4,Elavl2,Ezr,Fam129b,Ffar2,Fgf12,Fgf14,Fgfr4,Fzd9,Gata2,Gfi1,Gjd2,Gpr157,Gpr6,Grid1,Grik3,Grina,Hnf1a,Igsf9,Itpr3,Jak3,Kcne2,Kcnip1,Kcnk1,Kcnmb4,Mag,Map2k1,Mapt,Myc,Nell2,Nos3,Nr3c1,Nrxn1,Ntrk2,Palm,Pax8,Pcsk9,Pif1,Plod3,Por,Ppfia3,RGD1311558,Rab3a,Rbp4,S1pr1,Scn5a,Shank1,Siah1,Slc25a4,Slc39a3,Src,Stim1,Stpg1,Syt3,Tbx3,Trpm2,Unc13a,Usp7,Vwf
GO:0042391	regulation of membrane potential	23	291	0.00086	Adora2a,Ank3,Atp1a3,Cacna1a,Cacna1g,Cacna1i,Cacna2d1,Dbn1,Dlg4,Fgf12,Fgf14,Gjd2,Grid1,Grik3,Kcne2,Kcnip1,Kcnk1,Kcnmb4,Myc,Nrxn1,Scn5a,Shank1,Src
GO:0006996	organelle organization	58	1194	0.001	Actn1,Anp32b,Arf6,Arpc1a,Bet1,Bop1,Bsn,Capn3,Cbx7,Cdc20,Celsr3,Chd6,Cln8,Clvs2,Dapk3,Dbn1,Dlg4,Dnmt3a,Dnmt3l,Drc1,Dyrk3,Dyx1c1,Ezr,Grb7,Hipk4,Hnf1a,Kdm8,Map2k1,Mapt,Mical2,Msrb2,Myc,Nolc1,Nr3c1,Nrxn1,Odf2,Pacsin1,Palm,Pif1,Plec,RGD1311558,RGD1559696,Rab31,Rab3a,Rere,Rtn3,S1pr1,Sh3bp1,Shank1,Slc25a4,Sorbs2,Src,Stpg1,Synj2,Tppp3,Tubb5,Unc13a,Usp7
GO:0034765	regulation of ion transmembrane transport	23	295	0.001	Acsl5,Ank3,Cabp1,Cacna1a,Cacna1g,Cacna1i,Cacna2d1,Cacna2d2,Capn3,Dlg4,Fgf12,Fgf14,Kcne2,Kcng1,Kcnip1,Kcnip4,Kcnj4,Nrxn1,Pcsk9,Scn5a,Shank1,Slc25a4,Stim1
GO:1904062	regulation of cation transmembrane transport	18	194	0.0011	Ank3,Cabp1,Cacna1a,Cacna2d1,Capn3,Dlg4,Fgf12,Fgf14,Kcne2,Kcng1,Kcnip1,Kcnip4,Nrxn1,Pcsk9,Scn5a,Shank1,Slc25a4,Stim1
GO:0048468	cell development	38	661	0.0012	Adora2a,Bmp6,Capn3,Celsr2,Celsr3,Chst11,Dgkg,Dlg4,Dpysl5,Ezr,Gata2,Gdf6,Grm4,Igsf9,Limk2,Llgl1,Mag,Map2k1,Mapt,Nolc1,Ntrk2,Odf2,Pacsin1,Pcsk4,Plod3,RGD1311558,Rab3a,Rere,Sh3gl2,Shank1,Sorbs2,Src,Stk24,Tbx3,Tgfb3,Tgfbr2,Unc5a,Vapa
GO:0009887	animal organ morphogenesis	22	283	0.0015	Adam15,Bmp6,Chad,Chst11,Crip1,Gata2,Hnf1a,Inppl1,Mical2,Myc,Pax8,Plod3,Rogdi,S1pr1,Scn5a,Slc39a3,Src,Stim1,Tbx3,Tdrd7,Tgfb3,Tgfbr2
GO:0034762	regulation of transmembrane transport	25	350	0.0015	Acsl5,Ank3,Cabp1,Cacna1a,Cacna1g,Cacna1i,Cacna2d1,Cacna2d2,Capn3,Dlg4,Fgf12,Fgf14,Kcne2,Kcng1,Kcnip1,Kcnip4,Kcnj4,Myc,Nrxn1,Pcsk9,Pla2g1b,Scn5a,Shank1,Slc25a4,Stim1
GO:0051716	cellular response to stimulus	107	2719	0.0015	Acsl5,Adam15,Adora2a,Adra2c,Ank3,Arhgap27,Arhgef28,Arrb2,Atp1a3,Avp,Axin2,Bmp6,Brinp3,Cacna1g,Capn2,Capn3,Cd8a,Cebpe,Celsr2,Celsr3,Chad,Chd6,Crip1,Csnk1g2,Dapk3,Dbn1,Dgkg,Dnmt3a,Dok3,ENSRNOG00000050782,Elavl2,Ezr,Ffar2,Fgf12,Fgfr4,Fto,Fzd9,Galr3,Gdf6,Gfi1,Gnb2,Gng7,Gpr157,Gpr6,Grasp,Grb7,Grid1,Grik3,Grm4,Hgf,Hnf1a,Igfals,Itpr3,Jak3,Kcne2,Kcnj4,Limk2,Lrrtm4,Lzts2,Mag,Map2k1,Mapt,Matk,Mtr,Myc,Nos3,Nr3c1,Nrg2,Nrxn1,Ntrk2,Palm,Pax8,Pcsk9,Pif1,Pkm,Pla2g1b,Plod1,Plod3,Pomt1,Por,Ppp1r14a,Ppp1r15a,Prkaca,RGD1311558,Rab31,Rab3a,Rasgrp1,Rassf9,Rita1,S1pr1,Scn5a,Sh3bp1,Sh3gl2,Src,Sstr2,Stk24,Tbx3,Tgfb3,Tgfbr2,Tle3,Trpm2,Ucp3,Unc13a,Unc5a,Usp7,Vegfb,Vwf
GO:0023052	signaling	84	2006	0.0018	Adam15,Adora2a,Adra2c,Ank3,Arhgap27,Arhgef28,Arrb2,Atp1a3,Avp,Axin2,Bmp6,Cacna1a,Cacna1g,Cacna1i,Cd8a,Celsr2,Celsr3,Chad,Crip1,Csnk1g2,Dapk3,Dgkg,Dok3,ENSRNOG00000050782,Ezr,Fam129b,Ffar2,Fgf12,Fgfr4,Fzd9,Galr3,Gdf6,Gjd2,Gnb2,Gng7,Gpr157,Gpr6,Grasp,Grb7,Grid1,Grik3,Grm4,Hgf,Hnf1a,Itpr3,Jak3,Kcne2,Kcnmb4,Limk2,Lrrtm4,Lzts2,Map2k1,Mapt,Matk,Myc,Nos3,Nr3c1,Nrg2,Nrxn1,Ntrk2,Palm,Ppfia3,Ppp1r15a,RGD1311558,Rab3a,Rasgrp1,Rassf9,Rita1,S1pr1,Scn5a,Sh3bp1,Src,Sstr2,Stk24,Syt3,Tbx3,Tbx5,Tgfb3,Tgfbr2,Tle3,Trpm2,Unc13a,Unc5a,Vegfb
GO:0043269	regulation of ion transport	29	452	0.0019	Acsl5,Adora2a,Ank3,Arrb2,Avp,Cabp1,Cacna1a,Cacna1g,Cacna1i,Cacna2d1,Cacna2d2,Capn3,Dlg4,Fgf12,Fgf14,Jak3,Kcne2,Kcng1,Kcnip1,Kcnip4,Kcnj4,Nr3c1,Nrxn1,Pcsk9,Scn5a,Shank1,Slc15a1,Slc25a4,Stim1

GO:0071840	cellular component organization or biogenesis	86	2085	0.0023	Actn1,Adora2a,Aldh1a3,Ank3,Anp32b,Arf6,Arpc1a,Bet1,Bop1,Bsn,Capn3,Cbx7,Ccdc80,Cdc20,Celsr2,Celsr3,Chd6,Cln8,Clvs2,Dapk3,Dbn1,Diexf,Dkc1,Dlg4,Dnmt3a,Dnmt3l,Dpysl5,Drc1,Dyrk3,Dyx1c1,Ezr,Grb7,Grm4,Hgf,Hipk4,Hnf1a,Igsf9,Itpr3,Kcng1,Kdm8,Llgl1,Lrrtm4,Mag,Map2k1,Mapt,Mical2,Msrb2,Myc,Nell2,Nolc1,Nr3c1,Nrxn1,Odf2,Pacsin1,Palm,Pif1,Pkm,Plec,Plod3,Pomt1,RGD1311558,RGD1559696,Rab31,Rab3a,Rasgrp1,Rere,Rpl35a,Rtn3,S1pr1,Sh3bp1,Sh3gl2,Shank1,Slc25a4,Slc39a3,Sorbs2,Src,Stk24,Stpg1,Synj2,Tgfb3,Tppp3,Tubb5,Unc13a,Unc5a,Usp7,Vapa
GO:2001257	regulation of cation channel activity	12	98	0.0023	Ank3,Cabp1,Cacna1a,Cacna2d1,Dlg4,Fgf12,Fgf14,Kcne2,Kcng1,Nrxn1,Shank1,Stim1
GO:0007010	cytoskeleton organization	26	391	0.0027	Actn1,Arf6,Arpc1a,Bsn,Capn3,Cdc20,Cln8,Dbn1,Drc1,Dyx1c1,Ezr,Mapt,Mical2,Msrb2,Odf2,Pacsin1,Palm,Plec,RGD1311558,S1pr1,Sh3bp1,Shank1,Sorbs2,Src,Tppp3,Tubb5
GO:0000902	cell morphogenesis	19	236	0.0029	Adora2a,Celsr2,Celsr3,Dlg4,Dpysl5,Hgf,Llgl1,Map2k1,Pacsin1,Plod3,RGD1311558,Rab3a,Rere,Sh3gl2,Shank1,Slc39a3,Src,Stk24,Unc5a
GO:0016043	cellular component organization	83	2018	0.0032	Actn1,Adora2a,Aldh1a3,Ank3,Anp32b,Arf6,Arpc1a,Bet1,Bop1,Bsn,Capn3,Cbx7,Ccdc80,Cdc20,Celsr2,Celsr3,Chd6,Cln8,Clvs2,Dapk3,Dbn1,Dlg4,Dnmt3a,Dnmt3l,Dpysl5,Drc1,Dyrk3,Dyx1c1,Ezr,Grb7,Grm4,Hgf,Hipk4,Hnf1a,Igsf9,Itpr3,Kcng1,Kdm8,Llgl1,Lrrtm4,Mag,Map2k1,Mapt,Mical2,Msrb2,Myc,Nell2,Nolc1,Nr3c1,Nrxn1,Odf2,Pacsin1,Palm,Pif1,Pkm,Plec,Plod3,Pomt1,RGD1311558,RGD1559696,Rab31,Rab3a,Rasgrp1,Rere,Rtn3,S1pr1,Sh3bp1,Sh3gl2,Shank1,Slc25a4,Slc39a3,Sorbs2,Src,Stk24,Stpg1,Synj2,Tgfb3,Tppp3,Tubb5,Unc13a,Unc5a,Usp7,Vapa
GO:0007154	cell communication	85	2085	0.0033	Adam15,Adora2a,Adra2c,Ank3,Arhgap27,Arhgef28,Arrb2,Atp1a3,Avp,Axin2,Bmp6,Cacna1a,Cacna1g,Cacna1i,Cd8a,Celsr2,Celsr3,Chad,Crip1,Csnk1g2,Dapk3,Dgkg,Dok3,ENSRNOG00000050782,Ezr,Fam129b,Ffar2,Fgf12,Fgfr4,Fzd9,Galr3,Gdf6,Gjd2,Gnb2,Gng7,Gpr157,Gpr6,Grasp,Grb7,Grid1,Grik3,Grm4,Hgf,Hnf1a,Itpr3,Jak3,Kcnmb4,Limk2,Lrrtm4,Lzts2,Map2k1,Mapt,Matk,Mtr,Myc,Nos3,Nr3c1,Nrg2,Nrxn1,Ntrk2,Palm,Pcsk9,Ppfia3,Ppp1r15a,RGD1311558,Rab3a,Rasgrp1,Rassf9,Rita1,S1pr1,Scn5a,Sh3bp1,Src,Sstr2,Stk24,Syt3,Tbx3,Tbx5,Tgfb3,Tgfbr2,Tle3,Trpm2,Unc13a,Unc5a,Vegfb
GO:0042127	regulation of cell population proliferation	39	730	0.0033	Adk,Adora2a,Anp32b,Avp,Axin2,Bmp6,Cdc20,Chst11,Dkc1,Fam129b,Fgfr4,Fzd9,Gata2,Grm4,Hgf,Jak3,Matk,Myc,Nolc1,Nos3,Nr3c1,Ntrk2,Pla2g1b,Por,Ppp1cc,Ppp1r15a,Prkaca,Rnf187,Rogdi,S1pr1,Scn5a,Src,Sstr2,Tbx3,Tbx5,Tes,Tgfb3,Tgfbr2,Vegfb
GO:0022898	regulation of transmembrane transporter activity	14	142	0.004	Ank3,Cabp1,Cacna1a,Cacna2d1,Dlg4,Fgf12,Fgf14,Kcne2,Kcng1,Nrxn1,Pcsk9,Shank1,Slc25a4,Stim1
GO:0048583	regulation of response to stimulus	69	1609	0.0043	Adk,Adora2a,Adra2c,Arf6,Arhgef28,Arrb2,Avp,Axin2,Bmp6,Bop1,C4a,Cabp1,Capn3,Chad,Chst11,Dapk3,Dbn1,Dlg4,Dyrk3,Dyx1c1,Ezr,Fbxl15,Ffar2,Fgf12,Fgf14,Fgfr4,Fzd9,Gata2,Gdf6,Gfi1,Grb7,Grina,Hgf,Hipk4,Hnf1a,Igfbp6,Inppl1,Jak3,Lfng,Lrrtm4,Map2k1,Masp1,Mefv,Myc,Nr3c1,Nrg2,Nrxn1,Ntrk2,Palm,Pcsk9,Pgc,Por,Psd,Rasgrp1,Rita1,S1pr1,Scn5a,Sh3bp1,Shank1,Siah1,Slc25a4,Spred2,Src,Stk24,Tgfb3,Tgfbr2,Trim63,Trpm2,Vegfb
GO:0051239	regulation of multicellular organismal process	60	1346	0.0048	Adk,Adora2a,Adra2c,Arf6,Arrb2,Avp,Axin2,Bmp6,Brinp3,Cacna1g,Cacna2d2,Capn3,Cdc20,Celsr3,Dbn1,Dlg4,Elavl2,Ezr,Ffar2,Fgf12,Fkbpl,Fto,Fzd9,Gata2,Gdf6,Gfi1,Hgf,Inppl1,Jak3,Kcne2,Lfng,Mag,Map2k1,Mapt,Mefv,Myc,Nr3c1,Nrxn1,Ntrk2,Pacsin1,Pax8,Pcsk9,Por,Ppp1cc,Ppp1r15a,RGD1311558,Rasgrp1,S1pr1,Scn5a,Shank1,Slc25a4,Src,Sstr2,Stk24,Tbx3,Tbx5,Tgfb3,Tgfbr2,Trim63,Vegfb
GO:0098742	cell-cell adhesion via plasma-membrane adhesion molecules	10	75	0.0048	Cdh22,Celsr2,Celsr3,Fat3,Gypc,Igsf9,Mag,Mmp24,Nrxn1,Tgfbr2
GO:2000026	regulation of multicellular organismal development	44	889	0.0052	Adra2c,Arf6,Arrb2,Axin2,Bmp6,Brinp3,Capn3,Cdc20,Celsr3,Dbn1,Dlg4,Elavl2,Fkbpl,Fzd9,Gata2,Gdf6,Gfi1,Hgf,Inppl1,Jak3,Lfng,Mag,Mapt,Myc,Nr3c1,Nrxn1,Ntrk2,Pacsin1,Pax8,Por,Ppp1cc,Ppp1r15a,RGD1311558,Rasgrp1,S1pr1,Shank1,Slc25a4,Src,Stk24,Tbx3,Tbx5,Tgfb3,Tgfbr2,Vegfb
GO:0006810	transport	75	1810	0.0053	Abcd2,Acsl5,Anp32b,Anxa6,Arf6,Arhgap27,Arrb2,Asic4,Atp1a3,Bet1,Bsn,Cacna1a,Cacna1g,Cacna1i,Cacna2d1,Cacna2d2,Cebpe,Cln8,Csnk1g2,Ezr,Fam129b,Gata2,Grid1,Grik3,Grina,Grm4,Hare,Hnf1a,Itpr3,Kcne2,Kcng1,Kcnip1,Kcnip4,Kcnj4,Kcnk1,Kcnmb4,Klc1,Llgl1,Map2k1,Myc,Nrxn1,Pacsin1,Pcsk9,Pip5k1c,Pla2g1b,Ppfia3,Psd,RGD1559696,Rab31,Rab3a,Rasgrp1,Rassf9,Rbp4,Rita1,Rtn3,Scamp4,Scn5a,Sh3bp1,Sh3gl2,Slc10a4,Slc15a1,Slc25a4,Slc39a3,Slc3a2,Src,Stim1,Ston2,Stpg1,Svop,Syt3,Tbx3,Tgfbr2,Trpm2,Ucp3,Unc13a
GO:0032989	cellular component morphogenesis	20	275	0.0056	Adora2a,Capn3,Celsr2,Celsr3,Dlg4,Dpysl5,Hgf,Llgl1,Map2k1,Pacsin1,Plod3,RGD1311558,Rab3a,Rere,Sh3gl2,Shank1,Slc39a3,Src,Stk24,Unc5a
GO:0098609	cell-cell adhesion	13	130	0.0056	Adam15,Cdh22,Celsr2,Celsr3,Ezr,Fat3,Gypc,Igsf9,Mag,Mmp24,Nrxn1,Src,Tgfbr2
GO:0070887	cellular response to chemical stimulus	60	1365	0.0061	Acsl5,Adam15,Ank3,Arrb2,Atp1a3,Axin2,Bmp6,Brinp3,Cacna1g,Capn2,Capn3,Cebpe,Chad,Chd6,Crip1,Dapk3,Dbn1,Dnmt3a,Elavl2,Ezr,Ffar2,Fgf12,Fgfr4,Gdf6,Gfi1,Hgf,Igfals,Itpr3,Jak3,Kcne2,Lrrtm4,Mapt,Mtr,Myc,Nos3,Nr3c1,Nrxn1,Ntrk2,Pax8,Pcsk9,Pkm,Pla2g1b,Plod1,Plod3,Pomt1,Por,Ppp1r14a,Ppp1r15a,Rab31,S1pr1,Scn5a,Sh3gl2,Src,Sstr2,Stk24,Tgfb3,Tgfbr2,Trpm2,Ucp3,Vwf
GO:0051234	establishment of localization	76	1856	0.0062	Abcd2,Acsl5,Anp32b,Anxa6,Arf6,Arhgap27,Arrb2,Asic4,Atp1a3,Bet1,Bsn,Cacna1a,Cacna1g,Cacna1i,Cacna2d1,Cacna2d2,Cebpe,Cln8,Csnk1g2,Dlg4,Ezr,Fam129b,Gata2,Grid1,Grik3,Grina,Grm4,Hare,Hnf1a,Itpr3,Kcne2,Kcng1,Kcnip1,Kcnip4,Kcnj4,Kcnk1,Kcnmb4,Klc1,Llgl1,Map2k1,Myc,Nrxn1,Pacsin1,Pcsk9,Pip5k1c,Pla2g1b,Ppfia3,Psd,RGD1559696,Rab31,Rab3a,Rasgrp1,Rassf9,Rbp4,Rita1,Rtn3,Scamp4,Scn5a,Sh3bp1,Sh3gl2,Slc10a4,Slc15a1,Slc25a4,Slc39a3,Slc3a2,Src,Stim1,Ston2,Stpg1,Svop,Syt3,Tbx3,Tgfbr2,Trpm2,Ucp3,Unc13a
GO:0003008	system process	41	823	0.0071	Adora2a,Adra2c,Arrb2,Atp1a3,Avp,Cabp1,Cacna1a,Cacna1g,Cacna1i,Cln8,Dbn1,Dlg4,Elavl2,Ezr,Fam129b,Fgf12,Fzd9,Gjd2,Grid1,Grik3,Hnf1a,Kcne2,Kcnmb4,Map2k1,Mapt,Mmp24,Nos3,Nr3c1,Ntrk2,Plod3,Pomk,Rab3a,Rbp4,S1pr1,Scn5a,Shank1,Sorbs2,Src,Sstr2,Tbx3,Trim63
GO:0007267	cell-cell signaling	27	455	0.0081	Adora2a,Adra2c,Axin2,Cacna1a,Cacna1g,Celsr3,Csnk1g2,Fam129b,Fgf12,Fzd9,Gjd2,Grid1,Grik3,Grm4,Hgf,Hnf1a,Kcnmb4,Lzts2,Myc,Ppfia3,Rab3a,Scn5a,Syt3,Tbx3,Tbx5,Tle3,Unc13a
GO:0007015	actin filament organization	11	101	0.0087	Actn1,Arpc1a,Dbn1,Ezr,Mical2,Msrb2,Pacsin1,RGD1311558,Sh3bp1,Sorbs2,Src

GO:0032412	regulation of ion transmembrane transporter activity	13	138	0.0087	Ank3,Cabp1,Cacna1a,Cacna2d1,Dlg4,Fgf12,Fgf14,Kcne2,Kcng1,Nrxn1,Pcsk9,Shank1,Stim1
GO:0042221	response to chemical	88	2261	0.0087	Acsl5,Adam15,Adora2a,Aldh1a3,Ank3,Arrb2,Atp1a3,Avp,Axin2,Bmp6,Brinp3,Cacna1g,Capn2,Capn3,Cbx7,Cebpe,Celsr3,Chad,Chd6,Cln8,Crip1,Cyb5a,Dapk3,Dbn1,Dnmt3a,Dnmt3l,Dpysl5,Elavl2,Ezr,Ffar2,Fgf12,Fgfr4,Gata2,Gdf6,Gfi1,Hgf,Hnf1a,Igfals,Inppl1,Itpr3,Jak3,Kcne2,Kcnk1,Kcnmb4,Lrrtm4,Map2k1,Mapt,Mefv,Msi1,Mtr,Myc,Nos3,Nr3c1,Nrxn1,Ntrk2,Pax8,Pcsk9,Pip5k1c,Pkm,Pla2g1b,Plec,Plod1,Plod3,Pomt1,Por,Ppp1r14a,Ppp1r15a,Rab31,Rbp4,S1pr1,Scn5a,Sh3gl2,Shank1,Slc25a4,Src,Sstr2,Stim1,Stk24,Syt3,Tgfb3,Tgfbr2,Tox2,Trim63,Trpm2,Ucp3,Unc5a,Vegfb,Vwf
**GO:0048812**	**neuron projection morphogenesis**	**15**	**179**	**0.0088**	**Adora2a,Celsr2,Celsr3,Dlg4,Dpysl5,Llgl1,Map2k1,Pacsin1,RGD1311558,Rab3a,Rere,Sh3gl2,Shank1,Stk24,Unc5a**
GO:0009966	regulation of signal transduction	55	1248	0.0091	Adora2a,Adra2c,Arf6,Arhgef28,Arrb2,Avp,Axin2,Bmp6,Bop1,Cabp1,Capn3,Chad,Chst11,Dapk3,Dbn1,Dlg4,Dyrk3,Dyx1c1,Ezr,Fbxl15,Fgf12,Fgf14,Fgfr4,Fzd9,Gata2,Gdf6,Gfi1,Grb7,Grina,Hgf,Hipk4,Hnf1a,Igfbp6,Inppl1,Lfng,Lrrtm4,Map2k1,Nr3c1,Nrg2,Nrxn1,Ntrk2,Palm,Pcsk9,Por,Psd,Rasgrp1,Rita1,Sh3bp1,Shank1,Siah1,Spred2,Src,Stk24,Tgfb3,Vegfb
GO:0010959	regulation of metal ion transport	18	246	0.0091	Adora2a,Ank3,Arrb2,Cabp1,Cacna1a,Cacna1g,Cacna2d1,Capn3,Fgf12,Fgf14,Jak3,Kcne2,Kcng1,Kcnip1,Kcnip4,Pcsk9,Scn5a,Stim1
GO:0021700	developmental maturation	10	85	0.0091	Dlg4,Gata2,Nrxn1,Palm,Pcsk4,Rab3a,Rere,S1pr1,Shank1,Unc13a
GO:0022414	reproductive process	36	703	0.0091	Adam15,Adra2c,Arrb2,Atat1,Avp,B4galnt1,Bmp6,Cacna1i,Cbx7,Celsr2,Crip1,Dbn1,Dnmt3a,Dnmt3l,Gata2,Hnf1a,Lfng,Limk2,Mapt,Myc,Nell2,Nos3,Nr3c1,Odf2,Pcsk4,Plec,Scamp4,Siah1,Src,Sstr2,Tbx3,Tdrd7,Tgfb3,Tgfbr2,Tox2,Trpm2
GO:0030029	actin filament-based process	17	223	0.0091	Actn1,Arf6,Arpc1a,Cacna1g,Capn3,Dbn1,Ezr,Kcne2,Mical2,Msrb2,Pacsin1,RGD1311558,S1pr1,Scn5a,Sh3bp1,Sorbs2,Src
GO:0050793	regulation of developmental process	50	1096	0.0091	Adra2c,Arf6,Arrb2,Atat1,Axin2,Bmp6,Brinp3,Capn3,Cdc20,Celsr3,Csnk1g2,Dapk3,Dbn1,Dlg4,Elavl2,Ezr,Fkbpl,Fto,Fzd9,Gata2,Gdf6,Gfi1,Hgf,Inppl1,Jak3,Lfng,Mag,Mapt,Myc,Nr3c1,Nrxn1,Ntrk2,Pacsin1,Palm,Pax8,Por,Ppp1cc,Ppp1r15a,RGD1311558,Rasgrp1,S1pr1,Shank1,Slc25a4,Src,Stk24,Tbx3,Tbx5,Tgfb3,Tgfbr2,Vegfb
**GO:0050804**	**modulation of chemical synaptic transmission**	**19**	**268**	**0.0091**	**Adora2a,Arrb2,Bcan,Cabp1,Cdc20,Dbn1,Dlg4,Fgf14,Grik3,Grm4,Mapt,Nr3c1,Nrxn1,Ntrk2,Ppfia3,Prkaca,Rab3a,Shank1,Unc13a**
**GO:0050905**	**neuromuscular process**	**7**	**39**	**0.0091**	**Adora2a,Cln8,Dbn1,Dlg4,Fgf12,Pomk,Shank1**
GO:0045935	positive regulation of nucleobase-containing compound metabolic process	34	654	0.0099	Arrb2,Bmp6,Capn3,Cbx7,Cebpe,Chd6,Dnmt3l,Fam129b,Fgfr4,Galr3,Gata2,Gdf6,Hgf,Hnf1a,Kdm8,Map2k1,Mical2,Myc,Nfe2,Nolc1,Nos3,Nr3c1,Pax8,Phox2a,Rere,Rnf187,S1pr1,Src,Tbx3,Tbx5,Tgfb3,Tox2,Usp7,Zfp90
GO:0048523	negative regulation of cellular process	75	1880	0.011	Adam15,Adora2a,Ank3,Anp32b,Arf6,Arrb2,Avp,Axin2,Bmp6,Brinp3,C4a,Cabp1,Capn3,Cbx7,Chad,Chst11,Cln8,Dapk3,Dbn1,Dlg4,Dnmt3a,Dnmt3l,Dyrk3,Ezr,Fam129b,Foxg1,Fzd9,Gata2,Gfi1,Grb7,Grik3,Grina,Grm4,Hgf,Hnf1a,Inppl1,Itpr3,Jak3,Kcne2,Lfng,Lrrtm4,Mag,Map2k1,Mefv,Myc,Nos3,Nrxn1,Ntrk2,Palm,Pax8,Pcsk9,Pif1,Por,Ppp1r14a,Ppp1r15a,Prkaca,Rere,Rita1,S1pr1,Sh3bp1,Sh3rf2,Shank1,Slc25a4,Src,Sstr2,Stk24,Tbx3,Tbx5,Tes,Tgfb3,Tgfbr2,Trpm2,Tsks,Zbtb42,Zfp90
GO:0120036	plasma membrane bounded cell projection organization	26	449	0.0116	Adora2a,Arf6,Celsr2,Celsr3,Dlg4,Dpysl5,Drc1,Dyx1c1,Ezr,Grm4,Igsf9,Llgl1,Map2k1,Mapt,Odf2,Pacsin1,RGD1311558,Rab3a,Rere,S1pr1,Sh3bp1,Sh3gl2,Shank1,Stk24,Unc5a,Vapa
GO:0060255	regulation of macromolecule metabolic process	84	2174	0.012	Adk,Adora2a,Adra2c,Ank3,Anp32b,Arrb2,Avp,Axin2,Bmp6,C4a,Capn3,Cbx7,Cdc20,Cebpe,Celsr3,Chad,Chd6,Crip1,Csdc2,Dapk3,Dlg4,Dnmt3a,Dnmt3l,Dpf1,Dyx1c1,Elavl2,Ezr,Fam129b,Fbxl15,Fgfr4,Foxg1,Galr3,Gata2,Gdf6,Gfi1,Grb7,Hgf,Hnf1a,Inppl1,Jak3,Kcne2,Kdm8,Lrrtm4,Map2k1,Mbnl2,Mical2,Myc,Nfe2,Nolc1,Nr3c1,Ntrk2,Pax8,Pcsk9,Phox2a,Pif1,Pomt1,Por,Pou2f1,Ppp1cc,Ppp1r14a,Ppp1r15a,Rasgrp1,Rere,Rnf187,S1pr1,Sbno1,Sh3gl2,Sh3rf2,Src,Stk24,Taf6,Tbx3,Tbx5,Tdrd7,Tgfb3,Tgfbr2,Tle3,Tox2,Uncx,Usp7,Zbtb42,Zbtb8a,Zfp37,Zfp90
GO:0007166	cell surface receptor signaling pathway	38	775	0.0123	Adam15,Adora2a,Arrb2,Atp1a3,Axin2,Bmp6,Cd8a,Celsr2,Celsr3,Chad,Csnk1g2,Ffar2,Fgf12,Fgfr4,Fzd9,Gdf6,Gpr157,Grid1,Grik3,Grm4,Hgf,Hnf1a,Jak3,Lrrtm4,Lzts2,Matk,Myc,Nrg2,Ntrk2,RGD1311558,Rita1,Sh3bp1,Src,Tgfb3,Tgfbr2,Tle3,Unc5a,Vegfb
GO:0043170	macromolecule metabolic process	99	2669	0.0127	Adam15,Amz2,Arrb2,Atat1,Avp,B4galt4,Bop1,C4a,Capn11,Capn2,Capn3,Cbx7,Cdc20,Cebpe,Chd6,Chst11,Cln8,Cpa2,Csdc2,Csnk1g2,Ctrc,Dapk3,Diexf,Dkc1,Dnmt3a,Dnmt3l,Dpf1,Dyrk3,ENSRNOG00000050782,Ezr,Fbxl15,Fgfr4,Foxg1,Fto,Gata2,Gdf6,Gfi1,Hgf,Hipk4,Hnf1a,Jak3,Kdm8,Lfng,Limk2,Map2k1,Masp1,Matk,Mbnl2,Mmp24,Msrb2,Mtr,Myc,Nfe2,Nolc1,Nr3c1,Nrg2,Ntrk2,Pax8,Pcsk4,Pcsk9,Pgc,Phox2a,Pif1,Plod1,Plod3,Pomk,Pomt1,Por,Pou2f1,Ppp1cc,Prkaca,Qtrt1,Rab31,Repin1,Rere,Rnf182,Rnf187,Rnf44,Rpl35a,Sh3rf2,Siah1,Src,Stk24,Taf6,Tbx3,Tbx5,Tgfbr2,Tle3,Tox2,Trim63,Trmt11,U2af1l4,Uncx,Usp7,Zbtb42,Zbtb8a,Zdhhc22,Zfp37,Zfp90
**GO:0060322**	**head development**	**26**	**454**	**0.0129**	**Aldh1a3,Arrb2,Atat1,Atp1a3,Cdh22,Dbn1,Foxg1,Gata2,Inppl1,Limk2,Mapt,Myc,Nr3c1,Nrxn1,Ntrk2,Pomk,Rere,Rogdi,S1pr1,Scn5a,Src,Sstr2,Synj2,Tbx3,Tgfb3,Tgfbr2**
GO:0098657	import into cell	19	282	0.013	Arhgap27,Arrb2,Cebpe,Cln8,Csnk1g2,Ezr,Gata2,Hare,Kcne2,Kcnj4,Pacsin1,Pip5k1c,Rab31,Sh3bp1,Sh3gl2,Slc3a2,Ston2,Tgfbr2,Trpm2
GO:0006928	movement of cell or subcellular component	27	486	0.0144	Arrb2,Bsn,Cacna1g,Cacna1i,Celsr3,Dpysl5,Drc1,Dyx1c1,Ffar2,Fgfr4,Gata2,Hgf,Jak3,Kcne2,Map2k1,Ntrk2,Pomk,RGD1311558,RGD1559696,Rasgrp1,Rere,S1pr1,Scn5a,Sh3bp1,Src,Trpm2,Unc5a
GO:0007155	cell adhesion	20	309	0.0144	Adam15,Bcan,Cdh22,Celsr2,Celsr3,Ezr,Fat3,Gypc,Hare,Igfals,Igsf9,Inppl1,Mag,Mmp24,Ninj1,Nrxn1,Pip5k1c,Src,Tgfbr2,Vwf
**GO:0007417**	**central nervous system development**	**29**	**539**	**0.0144**	**Adora2a,Aldh1a3,Arrb2,Atat1,Atp1a3,Bcan,Cdh22,Cln8,Dbn1,Foxg1,Gata2,Inppl1,Mag,Mapt,Myc,Nr3c1,Nrxn1,Ntrk2,Pax8,Pomk,Rere,Rogdi,S1pr1,Scn5a,Src,Sstr2,Synj2,Tbx3,Tgfbr2**

GO:0008284	positive regulation of cell population proliferation	25	434	0.0144	Adk,Anp32b,Avp,Bmp6,Cdc20,Dkc1,Fgfr4,Fzd9,Gata2,Hgf,Jak3,Myc,Nolc1,Ntrk2,Pla2g1b,Ppp1cc,Prkaca,Rnf187,Rogdi,S1pr1,Scn5a,Tbx3,Tgfb3,Tgfbr2,Vegfb
GO:0009891	positive regulation of biosynthetic process	37	757	0.0144	Acsl5,Arrb2,Avp,Bmp6,Capn3,Cbx7,Cebpe,Chd6,Fam129b,Fgfr4,Galr3,Gata2,Gdf6,Hgf,Hnf1a,Jak3,Kdm8,Map2k1,Mical2,Myc,Nfe2,Nolc1,Nos3,Nr3c1,Pax8,Phox2a,Pomt1,Por,Rere,Rnf187,S1pr1,Src,Tbx3,Tbx5,Tgfb3,Tox2,Zfp90
GO:0031328	positive regulation of cellular biosynthetic process	36	728	0.0144	Acsl5,Arrb2,Avp,Bmp6,Capn3,Cbx7,Cebpe,Chd6,Fam129b,Fgfr4,Galr3,Gata2,Gdf6,Hgf,Hnf1a,Kdm8,Map2k1,Mical2,Myc,Nfe2,Nolc1,Nos3,Nr3c1,Pax8,Phox2a,Pomt1,Por,Rere,Rnf187,S1pr1,Src,Tbx3,Tbx5,Tgfb3,Tox2,Zfp90
GO:0048519	negative regulation of biological process	81	2096	0.0144	Adam15,Adora2a,Adra2c,Ank3,Anp32b,Arf6,Arrb2,Avp,Axin2,Bmp6,Brinp3,C4a,Cabp1,Capn3,Cbx7,Chad,Chst11,Cln8,Dapk3,Dbn1,Dlg4,Dnmt3a,Dnmt3l,Dyrk3,Ezr,Fam129b,Fgf12,Foxg1,Fzd9,Gata2,Gfi1,Grb7,Grik3,Grina,Grm4,Hgf,Hnf1a,Inppl1,Itpr3,Jak3,Kcne2,Lfng,Lrrtm4,Mag,Map2k1,Mefv,Myc,Nos3,Nrxn1,Ntrk2,Oasl,Palm,Pax8,Pcsk9,Pif1,Por,Pou2f1,Ppp1r14a,Ppp1r15a,Prkaca,Rere,Rita1,S1pr1,Sh3bp1,Sh3rf2,Shank1,Slc15a1,Slc25a4,Src,Sstr2,Stk24,Tbx3,Tbx5,Tes,Tgfb3,Tgfbr2,Trim63,Trpm2,Tsks,Zbtb42,Zfp90
GO:0043270	positive regulation of ion transport	15	196	0.0145	Acsl5,Adora2a,Ank3,Arrb2,Avp,Cacna2d1,Capn3,Fgf12,Fgf14,Jak3,Kcne2,Nr3c1,Scn5a,Slc25a4,Stim1
GO:0051094	positive regulation of developmental process	34	676	0.0145	Adra2c,Arrb2,Bmp6,Brinp3,Capn3,Cdc20,Dbn1,Dlg4,Elavl2,Ezr,Fzd9,Gata2,Gdf6,Hgf,Mag,Mapt,Myc,Nr3c1,Nrxn1,Ntrk2,Pacsin1,Pax8,Por,Ppp1cc,RGD1311558,Rasgrp1,Shank1,Slc25a4,Src,Stk24,Tbx5,Tgfb3,Tgfbr2,Vegfb
GO:0019222	regulation of metabolic process	89	2365	0.0146	Acsl5,Adk,Adora2a,Adra2c,Ank3,Anp32b,Arrb2,Avp,Axin2,Bmp6,C4a,Capn3,Cbx7,Cdc20,Cebpe,Celsr3,Chad,Chd6,Crip1,Csdc2,Dapk3,Dlg4,Dnmt3a,Dnmt3l,Dpf1,Dyx1c1,Elavl2,Ezr,Fam129b,Fbxl15,Fgfr4,Foxg1,Galr3,Gata2,Gdf6,Gfi1,Grb7,Hgf,Hnf1a,Inppl1,Jak3,Kcne2,Kdm8,Lrrtm4,Map2k1,Mbnl2,Mefv,Mical2,Myc,Nfe2,Nolc1,Nos3,Nr3c1,Ntrk2,Pax8,Pcsk9,Phox2a,Pif1,Pomt1,Por,Pou2f1,Ppp1cc,Ppp1r14a,Ppp1r15a,Prkaca,Rasgrp1,Rere,Rnf187,S1pr1,Sbno1,Sh3gl2,Sh3rf2,Src,Stk24,Taf6,Tbx3,Tbx5,Tdrd7,Tgfb3,Tgfbr2,Tle3,Tox2,Tsks,Uncx,Usp7,Zbtb42,Zbtb8a,Zfp37,Zfp90
**GO:0051962**	**positive regulation of nervous system development**	**21**	**336**	**0.0146**	**Adra2c,Bmp6,Brinp3,Cdc20,Dbn1,Dlg4,Elavl2,Gata2,Gdf6,Hgf,Mag,Mapt,Myc,Nr3c1,Nrxn1,Ntrk2,Pacsin1,Ppp1cc,RGD1311558,Shank1,Stk24**
GO:0071495	cellular response to endogenous stimulus	36	735	0.0149	Acsl5,Arrb2,Atp1a3,Bmp6,Cacna1g,Capn2,Dbn1,Dnmt3a,Elavl2,Ezr,Fgf12,Fgfr4,Gdf6,Igfals,Itpr3,Mtr,Myc,Nos3,Nr3c1,Ntrk2,Pax8,Pcsk9,Pkm,Pla2g1b,Plod1,Plod3,Por,Ppp1r15a,Rab31,Sh3gl2,Src,Sstr2,Tgfb3,Tgfbr2,Trpm2,Ucp3
GO:0007165	signal transduction	72	1825	0.0158	Adam15,Adora2a,Adra2c,Ank3,Arhgap27,Arhgef28,Arrb2,Atp1a3,Avp,Axin2,Bmp6,Cd8a,Celsr2,Celsr3,Chad,Crip1,Csnk1g2,Dapk3,Dgkg,Dok3,ENSRNOG00000050782,Ezr,Ffar2,Fgf12,Fgfr4,Fzd9,Galr3,Gdf6,Gnb2,Gng7,Gpr157,Gpr6,Grasp,Grb7,Grid1,Grik3,Grm4,Hgf,Hnf1a,Itpr3,Jak3,Limk2,Lrrtm4,Lzts2,Map2k1,Mapt,Matk,Myc,Nos3,Nr3c1,Nrg2,Nrxn1,Ntrk2,Palm,Ppp1r15a,RGD1311558,Rab3a,Rasgrp1,Rassf9,Rita1,S1pr1,Sh3bp1,Src,Sstr2,Stk24,Tgfb3,Tgfbr2,Tle3,Trpm2,Unc13a,Unc5a,Vegfb
**GO:0048666**	**neuron development**	**22**	**364**	**0.0158**	**Adora2a,Celsr2,Celsr3,Dgkg,Dlg4,Dpysl5,Gata2,Grm4,Igsf9,Llgl1,Map2k1,Mapt,Ntrk2,Pacsin1,RGD1311558,Rab3a,Rere,Sh3gl2,Shank1,Stk24,Unc5a,Vapa**
GO:0048732	gland development	18	267	0.0158	Aldh1a3,Arf6,Cbx7,Crip1,Gata2,Hgf,Hnf1a,Myc,Pax8,Pcsk9,Pkm,Pou2f1,Slc25a4,Src,Tbx3,Tgfb3,Tgfbr2,Vwf
GO:0051240	positive regulation of multicellular organismal process	39	823	0.0158	Adk,Adora2a,Adra2c,Arrb2,Avp,Bmp6,Brinp3,Cdc20,Dbn1,Dlg4,Elavl2,Ezr,Ffar2,Fzd9,Gata2,Gdf6,Hgf,Mag,Map2k1,Mapt,Myc,Nr3c1,Nrxn1,Ntrk2,Pacsin1,Pax8,Por,Ppp1cc,RGD1311558,Rasgrp1,Scn5a,Shank1,Slc25a4,Src,Stk24,Tbx5,Tgfb3,Tgfbr2,Vegfb
GO:0097435	supramolecular fiber organization	14	177	0.0158	Actn1,Arpc1a,Capn3,Dbn1,Ezr,Mical2,Msrb2,Pacsin1,Plod3,RGD1311558,Sh3bp1,Sorbs2,Src,Tppp3
GO:0010468	regulation of gene expression	60	1458	0.0172	Adk,Adora2a,Ank3,Arrb2,Avp,Bmp6,Capn3,Cbx7,Cebpe,Chd6,Crip1,Csdc2,Dapk3,Dnmt3a,Dpf1,Elavl2,Ezr,Fam129b,Fbxl15,Fgfr4,Foxg1,Galr3,Gata2,Gdf6,Gfi1,Grb7,Hnf1a,Kdm8,Map2k1,Mbnl2,Mical2,Myc,Nfe2,Nolc1,Nr3c1,Ntrk2,Pax8,Phox2a,Pou2f1,Ppp1cc,Ppp1r15a,Rere,Rnf187,S1pr1,Sbno1,Src,Taf6,Tbx3,Tbx5,Tdrd7,Tgfb3,Tgfbr2,Tle3,Tox2,Uncx,Usp7,Zbtb42,Zbtb8a,Zfp37,Zfp90
GO:0051050	positive regulation of transport	30	578	0.0172	Acsl5,Adora2a,Ank3,Arrb2,Avp,Bmp6,Cacna1g,Cacna1i,Cacna2d1,Capn3,Ezr,Ffar2,Fgf12,Fgf14,Gata2,Jak3,Kcne2,Nell2,Nr3c1,Pcsk9,Rab3a,Rasgrp1,Rbp4,Scn5a,Slc25a4,Src,Stim1,Syt3,Tgfb3,Trpm2
GO:2000112	regulation of cellular macromolecule biosynthetic process	55	1309	0.0183	Adora2a,Arrb2,Bmp6,Capn3,Cbx7,Cebpe,Chd6,Csdc2,Dapk3,Dnmt3a,Dpf1,Ezr,Fam129b,Fbxl15,Fgfr4,Foxg1,Galr3,Gata2,Gdf6,Gfi1,Grb7,Hgf,Hnf1a,Inppl1,Jak3,Kdm8,Map2k1,Mical2,Myc,Nfe2,Nolc1,Nr3c1,Pax8,Phox2a,Pif1,Pomt1,Pou2f1,Ppp1r15a,Rere,Rnf187,S1pr1,Sbno1,Src,Taf6,Tbx3,Tbx5,Tgfb3,Tle3,Tox2,Uncx,Usp7,Zbtb42,Zbtb8a,Zfp37,Zfp90
GO:0031326	regulation of cellular biosynthetic process	59	1434	0.0187	Acsl5,Adora2a,Arrb2,Avp,Bmp6,Capn3,Cbx7,Cebpe,Chd6,Csdc2,Dapk3,Dnmt3a,Dpf1,Ezr,Fam129b,Fbxl15,Fgfr4,Foxg1,Galr3,Gata2,Gdf6,Gfi1,Grb7,Hgf,Hnf1a,Inppl1,Jak3,Kdm8,Map2k1,Mical2,Myc,Nfe2,Nolc1,Nos3,Nr3c1,Pax8,Phox2a,Pif1,Pomt1,Por,Pou2f1,Ppp1r15a,Rere,Rnf187,S1pr1,Sbno1,Src,Taf6,Tbx3,Tbx5,Tgfb3,Tle3,Tox2,Uncx,Usp7,Zbtb42,Zbtb8a,Zfp37,Zfp90
GO:0071310	cellular response to organic substance	49	1129	0.0187	Acsl5,Adam15,Arrb2,Atp1a3,Axin2,Bmp6,Brinp3,Cacna1g,Capn2,Cebpe,Chad,Dapk3,Dbn1,Dnmt3a,Elavl2,Ezr,Ffar2,Fgf12,Fgfr4,Gdf6,Gfi1,Hgf,Igfals,Itpr3,Jak3,Lrrtm4,Mtr,Myc,Nos3,Nr3c1,Ntrk2,Pax8,Pcsk9,Pkm,Pla2g1b,Plod1,Plod3,Pomt1,Por,Ppp1r15a,Rab31,Sh3gl2,Src,Sstr2,Tgfb3,Tgfbr2,Trpm2,Ucp3,Vwf

GO:0010557	positive regulation of macromolecule biosynthetic process	33	666	0.019	Arrb2,Bmp6,Capn3,Cbx7,Cebpe,Chd6,Fam129b,Fgfr4,Galr3,Gata2,Gdf6,Hgf,Hnf1a,Jak3,Kdm8,Map2k1,Mical2,Myc,Nfe2,Nolc1,Nr3c1,Pax8,Phox2a,Pomt1,Rere,Rnf187,S1pr1,Src,Tbx3,Tbx5,Tgfb3,Tox2,Zfp90
GO:0007204	positive regulation of cytosolic calcium ion concentration	14	183	0.0193	Avp,Cacna1a,Cacna1g,Cacna1i,Cacna2d1,Capn3,Dlg4,Gata2,Gpr157,Gpr6,Itpr3,Jak3,S1pr1,Trpm2
GO:0045597	positive regulation of cell differentiation	27	504	0.0196	Adra2c,Arrb2,Bmp6,Brinp3,Dbn1,Dlg4,Elavl2,Gata2,Gdf6,Hgf,Mag,Mapt,Myc,Nr3c1,Ntrk2,Pacsin1,Pax8,Por,Ppp1cc,RGD1311558,Rasgrp1,Shank1,Slc25a4,Stk24,Tbx5,Tgfb3,Tgfbr2
GO:0045893	positive regulation of transcription, DNA-templated	27	504	0.0196	Bmp6,Capn3,Cbx7,Cebpe,Chd6,Fam129b,Galr3,Gata2,Gdf6,Hnf1a,Kdm8,Map2k1,Mical2,Myc,Nfe2,Nolc1,Nr3c1,Pax8,Phox2a,Rnf187,S1pr1,Src,Tbx3,Tbx5,Tgfb3,Tox2,Zfp90
GO:2000278	regulation of DNA biosynthetic process	7	50	0.0202	Arrb2,Fam129b,Fgfr4,Hgf,Myc,Pif1,Src
**GO:0050769**	**positive regulation of neurogenesis**	**19**	**302**	**0.0211**	**Adra2c,Bmp6,Brinp3,Dbn1,Dlg4,Elavl2,Gata2,Gdf6,Hgf,Mag,Mapt,Myc,Nr3c1,Ntrk2,Pacsin1,Ppp1cc,RGD1311558,Shank1,Stk24**
GO:0009888	tissue development	30	591	0.0221	Adam15,Bmp6,Chad,Chst11,Crip1,Ezr,Fbxl15,Fzd9,Hgf,Hnf1a,Kazn,Lfng,Mical2,Msi1,Myc,Ninj1,Nolc1,Pax8,Pkm,Plec,Plod3,S1pr1,Sh3bp1,Sorbs2,Src,Stim1,Tbx3,Tbx5,Tdrd7,Tgfbr2
GO:0042981	regulation of apoptotic process	35	731	0.0221	Adora2a,Anp32b,Arrb2,Avp,Bmp6,Capn3,Chst11,Cln8,Dapk3,Dyrk3,Fam129b,Fzd9,Gata2,Gdf6,Grina,Grm4,Hgf,Jak3,Mag,Map2k1,Myc,Nos3,Ntrk2,Pax8,Pcsk9,Por,Ppp1r15a,Siah1,Slc25a4,Src,Stk24,Stpg1,Tbx3,Tgfb3,Usp7
GO:0031323	regulation of cellular metabolic process	83	2217	0.0224	Acsl5,Adora2a,Adra2c,Arrb2,Avp,Axin2,Bmp6,C4a,Capn3,Cbx7,Cdc20,Cebpe,Celsr3,Chad,Chd6,Csdc2,Dapk3,Dlg4,Dnmt3a,Dnmt3l,Dpf1,Dyx1c1,Elavl2,Ezr,Fam129b,Fbxl15,Fgfr4,Foxg1,Galr3,Gata2,Gdf6,Gfi1,Grb7,Hgf,Hnf1a,Inppl1,Jak3,Kcne2,Kdm8,Lrrtm4,Map2k1,Mbnl2,Mefv,Mical2,Myc,Nfe2,Nolc1,Nos3,Nr3c1,Ntrk2,Pax8,Pcsk9,Phox2a,Pif1,Pomt1,Por,Pou2f1,Ppp1r14a,Ppp1r15a,Prkaca,Rasgrp1,Rere,Rnf187,S1pr1,Sbno1,Sh3gl2,Sh3rf2,Src,Stk24,Taf6,Tbx3,Tbx5,Tgfb3,Tgfbr2,Tle3,Tox2,Tsks,Uncx,Usp7,Zbtb42,Zbtb8a,Zfp37,Zfp90
GO:0051171	regulation of nitrogen compound metabolic process	79	2091	0.0235	Adora2a,Adra2c,Anp32b,Arrb2,Avp,Axin2,Bmp6,C4a,Capn3,Cbx7,Cdc20,Cebpe,Celsr3,Chad,Chd6,Csdc2,Dapk3,Dlg4,Dnmt3a,Dnmt3l,Dpf1,Dyx1c1,Elavl2,Ezr,Fam129b,Fbxl15,Fgfr4,Foxg1,Galr3,Gata2,Gdf6,Gfi1,Grb7,Hgf,Hnf1a,Inppl1,Jak3,Kcne2,Kdm8,Lrrtm4,Map2k1,Mbnl2,Mical2,Myc,Nfe2,Nolc1,Nos3,Nr3c1,Ntrk2,Pax8,Pcsk9,Phox2a,Pif1,Pomt1,Por,Pou2f1,Ppp1r14a,Ppp1r15a,Rasgrp1,Rere,Rnf187,S1pr1,Sbno1,Sh3rf2,Src,Stk24,Taf6,Tbx3,Tbx5,Tgfb3,Tgfbr2,Tle3,Tox2,Uncx,Usp7,Zbtb42,Zbtb8a,Zfp37,Zfp90
GO:0019219	regulation of nucleobase-containing compound metabolic process	55	1333	0.0243	Adora2a,Arrb2,Bmp6,Capn3,Cbx7,Cebpe,Chd6,Csdc2,Dapk3,Dnmt3a,Dnmt3l,Dpf1,Elavl2,Ezr,Fam129b,Fbxl15,Fgfr4,Foxg1,Galr3,Gata2,Gdf6,Gfi1,Hgf,Hnf1a,Inppl1,Kdm8,Map2k1,Mbnl2,Mical2,Myc,Nfe2,Nolc1,Nos3,Nr3c1,Pax8,Phox2a,Pif1,Pou2f1,Rere,Rnf187,S1pr1,Sbno1,Src,Taf6,Tbx3,Tbx5,Tgfb3,Tle3,Tox2,Uncx,Usp7,Zbtb42,Zbtb8a,Zfp37,Zfp90
**GO:0031175**	**neuron projection development**	**19**	**307**	**0.0243**	**Adora2a,Celsr2,Celsr3,Dlg4,Dpysl5,Grm4,Igsf9,Llgl1,Map2k1,Mapt,Pacsin1,RGD1311558,Rab3a,Rere,Sh3gl2,Shank1,Stk24,Unc5a,Vapa**
GO:1901385	regulation of voltage-gated calcium channel activity	5	24	0.0243	Cabp1,Cacna1a,Cacna2d1,Fgf14,Kcne2
GO:1903508	positive regulation of nucleic acid-templated transcription	28	543	0.0248	Bmp6,Capn3,Cbx7,Cebpe,Chd6,Fam129b,Galr3,Gata2,Gdf6,Hnf1a,Kdm8,Map2k1,Mical2,Myc,Nfe2,Nolc1,Nr3c1,Pax8,Phox2a,Rere,Rnf187,S1pr1,Src,Tbx3,Tbx5,Tgfb3,Tox2,Zfp90
**GO:0007420**	**brain development**	**24**	**437**	**0.0252**	**Aldh1a3,Arrb2,Atat1,Atp1a3,Cdh22,Dbn1,Foxg1,Gata2,Inppl1,Mapt,Myc,Nr3c1,Nrxn1,Ntrk2,Pomk,Rere,Rogdi,S1pr1,Scn5a,Src,Sstr2,Synj2,Tbx3,Tgfbr2**
GO:0010720	positive regulation of cell development	20	334	0.0252	Adra2c,Bmp6,Brinp3,Dbn1,Dlg4,Elavl2,Gata2,Gdf6,Hgf,Mag,Mapt,Myc,Nr3c1,Ntrk2,Pacsin1,Ppp1cc,RGD1311558,Shank1,Slc25a4,Stk24
GO:0050801	ion homeostasis	24	437	0.0252	Ank3,Atp1a3,Avp,Bmp6,Cacna1a,Cacna1g,Cacna1i,Cacna2d1,Capn3,Dlg4,Fgf12,Fgfr4,Gata2,Gpr157,Gpr6,Grina,Itpr3,Jak3,Myc,S1pr1,Scn5a,Src,Stim1,Trpm2
GO:0010941	regulation of cell death	38	829	0.0258	Adora2a,Anp32b,Arrb2,Avp,Bmp6,Capn3,Chst11,Cln8,Dapk3,Dnmt3a,Dyrk3,Fam129b,Fzd9,Gata2,Gdf6,Grina,Grm4,Hgf,Jak3,Lzts2,Mag,Map2k1,Myc,Nos3,Ntrk2,Pax8,Pcsk9,Por,Ppp1r15a,Siah1,Slc25a4,Src,Stk24,Stpg1,Tbx3,Tgfb3,Trpm2,Usp7
**GO:0060074**	**synapse maturation**	**3**	**5**	**0.0258**	**Nrxn1,Palm,Shank1**
GO:0006875	cellular metal ion homeostasis	19	313	0.0276	Atp1a3,Avp,Bmp6,Cacna1a,Cacna1g,Cacna1i,Cacna2d1,Capn3,Dlg4,Gata2,Gpr157,Gpr6,Grina,Itpr3,Jak3,Myc,S1pr1,Stim1,Trpm2
GO:0051128	regulation of cellular component organization	46	1073	0.0283	Adam15,Arf6,Arpc1a,Arrb2,Atat1,Avp,Capn2,Cdc20,Csnk1g2,Dapk3,Dbn1,Dlg4,Elavl2,Ezr,Fgfr4,Fzd9,Gata2,Gfi1,Hgf,Igfbp6,Igsf9,Inppl1,Mag,Map2k1,Mapt,Mefv,Myc,Nr3c1,Nrxn1,Ntrk2,Pacsin1,Palm,Pcsk9,Pif1,RGD1311558,Rab3a,S1pr1,Sh3gl2,Shank1,Slc25a4,Src,Stk24,Ston2,Syt3,Tgfb3,Trpm2
**GO:0045666**	**positive regulation of neuron differentiation**	**16**	**242**	**0.0289**	**Adra2c,Bmp6,Brinp3,Dbn1,Dlg4,Elavl2,Gata2,Gdf6,Hgf,Mapt,Nr3c1,Ntrk2,Pacsin1,RGD1311558,Shank1,Stk24**

GO:0010628	positive regulation of gene expression	33	693	0.029	Ank3,Arrb2,Avp,Bmp6,Capn3,Cbx7,Cebpe,Chd6,Ezr,Fam129b,Fgfr4,Galr3,Gata2,Gdf6,Hnf1a,Kdm8,Map2k1,Mical2,Myc,Nfe2,Nolc1,Nr3c1,Ntrk2,Pax8,Phox2a,Rnf187,S1pr1,Src,Tbx3,Tbx5,Tgfb3,Tox2,Zfp90
GO:0030036	actin cytoskeleton organization	14	197	0.0301	Actn1,Arf6,Arpc1a,Capn3,Dbn1,Ezr,Mical2,Msrb2,Pacsin1,RGD1311558,S1pr1,Sh3bp1,Sorbs2,Src
GO:0048562	embryonic organ morphogenesis	8	73	0.0301	Chst11,Gata2,Mical2,Pax8,Slc39a3,Tbx3,Tgfb3,Tgfbr2
GO:0055065	metal ion homeostasis	20	342	0.0301	Ank3,Atp1a3,Avp,Bmp6,Cacna1a,Cacna1g,Cacna1i,Cacna2d1,Capn3,Dlg4,Gata2,Gpr157,Gpr6,Grina,Itpr3,Jak3,Myc,S1pr1,Stim1,Trpm2
GO:0007178	transmembrane receptor protein serine/threonine kinase signaling pathway	8	74	0.032	Arrb2,Bmp6,Gdf6,Hnf1a,Ntrk2,Src,Tgfb3,Tgfbr2
GO:0001763	morphogenesis of a branching structure	7	57	0.0328	Hgf,Myc,Pax8,Rere,Src,Tbx3,Tgfbr2
GO:0080090	regulation of primary metabolic process	80	2165	0.0335	Acsl5,Adora2a,Adra2c,Anp32b,Arrb2,Avp,Axin2,Bmp6,C4a,Capn3,Cbx7,Cdc20,Cebpe,Celsr3,Chad,Chd6,Csdc2,Dapk3,Dlg4,Dnmt3a,Dnmt3l,Dpf1,Dyx1c1,Elavl2,Ezr,Fam129b,Fbxl15,Fgfr4,Foxg1,Galr3,Gata2,Gdf6,Gfi1,Grb7,Hgf,Hnf1a,Inppl1,Jak3,Kcne2,Kdm8,Lrrtm4,Map2k1,Mbnl2,Mical2,Myc,Nfe2,Nolc1,Nos3,Nr3c1,Ntrk2,Pax8,Pcsk9,Phox2a,Pif1,Pomt1,Por,Pou2f1,Ppp1r14a,Ppp1r15a,Rasgrp1,Rere,Rnf187,S1pr1,Sbno1,Sh3rf2,Src,Stk24,Taf6,Tbx3,Tbx5,Tgfb3,Tgfbr2,Tle3,Tox2,Uncx,Usp7,Zbtb42,Zbtb8a,Zfp37,Zfp90
GO:0003376	sphingosine-1-phosphate receptor signaling pathway	3	6	0.0339	Ezr,Gpr6,S1pr1
GO:0055085	transmembrane transport	34	731	0.0339	Abcd2,Asic4,Atp1a3,Cacna1a,Cacna1g,Cacna1i,Cacna2d1,Cacna2d2,Cln8,Grid1,Grik3,Grina,Hnf1a,Itpr3,Kcne2,Kcng1,Kcnip1,Kcnip4,Kcnj4,Kcnk1,Kcnmb4,Myc,Psd,Rbp4,Scn5a,Slc10a4,Slc15a1,Slc25a4,Slc39a3,Slc3a2,Stim1,Svop,Trpm2,Ucp3
GO:0048705	skeletal system morphogenesis	7	58	0.0347	Bmp6,Chad,Chst11,Inppl1,Slc39a3,Tgfb3,Tgfbr2
GO:0007156	homophilic cell adhesion via plasma membrane adhesion molecules	6	42	0.035	Cdh22,Celsr2,Celsr3,Fat3,Gypc,Igsf9
GO:0043154	negative regulation of cysteine-type endopeptidase activity involved in apoptotic process	6	42	0.035	Adora2a,Arrb2,Avp,Hgf,Por,Src
GO:0033043	regulation of organelle organization	24	455	0.0355	Arf6,Arpc1a,Arrb2,Atat1,Avp,Capn2,Cdc20,Dapk3,Dbn1,Ezr,Gata2,Gfi1,Hgf,Map2k1,Mapt,Myc,Pif1,Rab3a,S1pr1,Shank1,Src,Syt3,Tgfb3,Trpm2
GO:0043066	negative regulation of apoptotic process	23	429	0.036	Adora2a,Anp32b,Arrb2,Avp,Capn3,Chst11,Cln8,Dyrk3,Fam129b,Fzd9,Gata2,Grina,Grm4,Hgf,Jak3,Mag,Map2k1,Ntrk2,Pax8,Por,Slc25a4,Src,Tbx3
GO:0006807	nitrogen compound metabolic process	108	3120	0.0378	Acsl5,Adam15,Adk,Amz2,Arrb2,Atat1,Avp,B4galnt1,B4galt4,Bop1,C4a,Capn11,Capn2,Capn3,Cbx7,Cdc20,Cebpe,Chd6,Chst11,Cln8,Cpa2,Csdc2,Csnk1g2,Ctrc,Dapk3,Diexf,Dkc1,Dlg4,Dnmt3a,Dnmt3l,Dpf1,Dyrk3,ENSRNOG00000050782,Fbxl15,Fgfr4,Foxg1,Fto,Gata2,Gdf6,Gfi1,Hal,Hgf,Hipk4,Hnf1a,Jak3,Kdm8,Lfng,Limk2,Map2k1,Masp1,Matk,Mbnl2,Mmp24,Msrb2,Mtr,Myc,Nfe2,Nmnat2,Nolc1,Nos3,Nr3c1,Nrg2,Ntrk2,Oplah,Pax8,Pcsk4,Pcsk9,Pgc,Phox2a,Pif1,Pkm,Pla2g1b,Plod1,Plod3,Pomk,Pomt1,Por,Pou2f1,Ppp1cc,Prkaca,Qtrt1,Repin1,Rere,Rnf182,Rnf187,Rnf44,Rpl35a,Sh3rf2,Siah1,Src,Stk24,Taf6,Tbx3,Tbx5,Tgfbr2,Tle3,Tmem14c,Tox2,Trim63,Trmt11,U2af1l4,Uncx,Usp7,Zbtb42,Zbtb8a,Zdhhc22,Zfp37,Zfp90
GO:0044057	regulation of system process	21	378	0.0378	Adk,Adora2a,Adra2c,Avp,Bmp6,Cacna1g,Cacna2d2,Dbn1,Dlg4,Fgf12,Hgf,Kcne2,Mag,Map2k1,Nr3c1,Nrxn1,Scn5a,Shank1,Slc25a4,Sstr2,Trim63
GO:0048701	embryonic cranial skeleton morphogenesis	4	16	0.0378	Chst11,Slc39a3,Tgfb3,Tgfbr2
**GO:0048167**	**regulation of synaptic plasticity**	**11**	**138**	**0.039**	**Adora2a,Bcan,Cabp1,Cdc20,Dlg4,Fgf14,Mapt,Nr3c1,Ppfia3,Rab3a,Unc13a**
GO:0072507	divalent inorganic cation homeostasis	17	278	0.0394	Ank3,Avp,Cacna1a,Cacna1g,Cacna1i,Cacna2d1,Capn3,Dlg4,Gata2,Gpr157,Gpr6,Grina,Itpr3,Jak3,S1pr1,Stim1,Trpm2
GO:0007368	determination of left/right symmetry	5	29	0.0399	Drc1,Dyx1c1,Mical2,Tbx3,Tgfbr2
GO:0035418	protein localization to synapse	5	29	0.0399	Anks1b,Dlg4,Klc1,Nrxn1,Shank1

GO:0006355	regulation of transcription, DNA-templated	47	1135	0.0413	Adora2a,Arrb2,Bmp6,Capn3,Cbx7,Cebpe,Chd6,Csdc2,Dapk3,Dnmt3a,Dpf1,Ezr,Fam129b,Fbxl15,Foxg1,Galr3,Gata2,Gdf6,Gfi1,Hnf1a,Kdm8,Map2k1,Mical2,Myc,Nfe2,Nolc1,Nr3c1,Pax8,Phox2a,Pou2f1,Rere,Rnf187,S1pr1,Sbno1,Src,Taf6,Tbx3,Tbx5,Tgfb3,Tle3,Tox2,Uncx,Usp7,Zbtb42,Zbtb8a,Zfp37,Zfp90
GO:0031325	positive regulation of cellular metabolic process	51	1259	0.0413	Acsl5,Adra2c,Arrb2,Avp,Axin2,Bmp6,Capn3,Cbx7,Cdc20,Cebpe,Chd6,Dlg4,Dnmt3l,Ezr,Fam129b,Fgfr4,Galr3,Gata2,Gdf6,Hgf,Hnf1a,Kcne2,Kdm8,Map2k1,Mefv,Mical2,Myc,Nfe2,Nolc1,Nos3,Nr3c1,Ntrk2,Pax8,Pcsk9,Phox2a,Pomt1,Por,Ppp1r15a,Rasgrp1,Rere,Rnf187,S1pr1,Src,Stk24,Tbx3,Tbx5,Tgfb3,Tgfbr2,Tox2,Usp7,Zfp90
GO:0044092	negative regulation of molecular function	25	490	0.0413	Adam15,Adora2a,Ank3,Arrb2,Avp,C4a,Cabp1,Chad,Dbn1,Fgf12,Galr3,Gfi1,Hgf,Inppl1,Kcne2,Lrrtm4,Palm,Pcsk9,Pif1,Por,Ppp1r14a,Sh3rf2,Src,Tsks,Zfp90
GO:0006897	endocytosis	14	209	0.0421	Arhgap27,Arrb2,Cebpe,Csnk1g2,Ezr,Gata2,Hare,Pacsin1,Pip5k1c,Rab31,Sh3bp1,Sh3gl2,Ston2,Tgfbr2
GO:0018108	peptidyl-tyrosine phosphorylation	8	80	0.0421	Dyrk3,Fgfr4,Jak3,Map2k1,Matk,Nrg2,Ntrk2,Src
GO:0032332	positive regulation of chondrocyte differentiation	3	7	0.0421	Bmp6,Gdf6,Por
**GO:0045664**	**regulation of neuron differentiation**	**21**	**384**	**0.0421**	**Adra2c,Arf6,Bmp6,Brinp3,Cdc20,Dbn1,Dlg4,Elavl2,Gata2,Gdf6,Gfi1,Hgf,Inppl1,Mag,Mapt,Nr3c1,Ntrk2,Pacsin1,RGD1311558,Shank1,Stk24**
GO:0051254	positive regulation of RNA metabolic process	28	575	0.0421	Bmp6,Capn3,Cbx7,Cebpe,Chd6,Fam129b,Galr3,Gata2,Gdf6,Hnf1a,Kdm8,Map2k1,Mical2,Myc,Nfe2,Nolc1,Nr3c1,Pax8,Phox2a,Rere,Rnf187,S1pr1,Src,Tbx3,Tbx5,Tgfb3,Tox2,Zfp90
GO:0051489	regulation of filopodium assembly	5	30	0.0421	Arf6,Dbn1,Nrxn1,Palm,Trpm2
GO:0071286	cellular response to magnesium ion	3	7	0.0421	Ank3,Nos3,Nr3c1
GO:0120035	regulation of plasma membrane bounded cell projection organization	21	386	0.0425	Arf6,Cdc20,Dbn1,Dlg4,Elavl2,Ezr,Gfi1,Hgf,Inppl1,Mag,Mapt,Nr3c1,Nrxn1,Ntrk2,Pacsin1,Palm,RGD1311558,Shank1,Src,Stk24,Trpm2
GO:1903532	positive regulation of secretion by cell	16	258	0.0425	Adora2a,Avp,Bmp6,Cacna1g,Cacna1i,Ezr,Ffar2,Gata2,Nell2,Nr3c1,Rab3a,Rasgrp1,Rbp4,Src,Tgfb3,Trpm2
GO:0006874	cellular calcium ion homeostasis	16	260	0.0452	Avp,Cacna1a,Cacna1g,Cacna1i,Cacna2d1,Capn3,Dlg4,Gata2,Gpr157,Gpr6,Grina,Itpr3,Jak3,S1pr1,Stim1,Trpm2
GO:0048638	regulation of developmental growth	12	166	0.0464	Capn3,Dbn1,Ezr,Fto,Mag,Mapt,Nr3c1,Por,RGD1311558,Slc25a4,Tbx5,Tgfbr2
GO:0043009	chordate embryonic development	12	167	0.0473	Capn2,Chst11,Gata2,Hnf1a,Lfng,Myc,Nolc1,Plod3,Slc39a3,Tbx3,Tgfb3,Tgfbr2
GO:0001501	skeletal system development	11	145	0.0478	Bcan,Bmp6,Chad,Chst11,Inppl1,Nos3,Slc39a3,Src,Tbx3,Tgfb3,Tgfbr2
GO:0043412	macromolecule modification	46	1121	0.0478	Arrb2,Atat1,Avp,B4galt4,Cdc20,Csnk1g2,Dapk3,Dkc1,Dnmt3a,Dnmt3l,Dyrk3,Fbxl15,Fgfr4,Fto,Gdf6,Hipk4,Hnf1a,Jak3,Kdm8,Lfng,Limk2,Map2k1,Matk,Mtr,Myc,Nrg2,Ntrk2,Plod1,Plod3,Pomk,Pomt1,Por,Ppp1cc,Prkaca,Qtrt1,Rnf182,Rnf187,Rnf44,Sh3rf2,Siah1,Src,Stk24,Tgfbr2,Trim63,Usp7,Zdhhc22
GO:0051173	positive regulation of nitrogen compound metabolic process	48	1184	0.0478	Adra2c,Arrb2,Avp,Axin2,Bmp6,Capn3,Cbx7,Cdc20,Cebpe,Chd6,Dlg4,Dnmt3l,Ezr,Fam129b,Fgfr4,Galr3,Gata2,Gdf6,Hgf,Hnf1a,Kcne2,Kdm8,Map2k1,Mical2,Myc,Nfe2,Nolc1,Nos3,Nr3c1,Ntrk2,Pax8,Pcsk9,Phox2a,Pomt1,Ppp1r15a,Rasgrp1,Rere,Rnf187,S1pr1,Src,Stk24,Tbx3,Tbx5,Tgfb3,Tgfbr2,Tox2,Usp7,Zfp90
GO:0060548	negative regulation of cell death	25	501	0.0478	Adora2a,Anp32b,Arrb2,Avp,Capn3,Chst11,Cln8,Dyrk3,Fam129b,Fzd9,Gata2,Grina,Grm4,Hgf,Jak3,Mag,Map2k1,Myc,Ntrk2,Pax8,Por,Ppp1r15a,Slc25a4,Src,Tbx3
GO:0044238	primary metabolic process	116	3438	0.0481	Abcd2,Acsf2,Acsl5,Adam15,Adk,Aldh1a3,Amz2,Arrb2,Atat1,Avp,B4galnt1,B4galt4,Bop1,C4a,Capn11,Capn2,Capn3,Cbx7,Cdc20,Cebpe,Chd6,Chst11,Cln8,Cpa2,Csdc2,Csnk1g2,Ctrc,Dapk3,Dgkg,Diexf,Dkc1,Dlg4,Dnmt3a,Dnmt3l,Dpf1,Dyrk3,ENSRNOG00000050782,Fbxl15,Fgfr4,Foxg1,Fto,Gata2,Gdf6,Gfi1,Hal,Hgf,Hipk4,Hnf1a,Inppl1,Jak3,Kdm8,Lfng,Limk2,Map2k1,Masp1,Matk,Mbnl2,Mmp24,Msrb2,Mtr,Myc,Nfe2,Nmnat2,Nolc1,Nr3c1,Nrg2,Ntrk2,Pax8,Pcsk4,Pcsk9,Pcyt2,Pgc,Phox2a,Pif1,Pip5k1c,Pkm,Pla2g1b,Plod1,Plod3,Pomk,Pomt1,Por,Pou2f1,Ppp1cc,Prkaca,Qtrt1,Rbp4,Repin1,Rere,Rnf182,Rnf187,Rnf44,Rpl35a,Sh3rf2,Siah1,Slc3a2,Src,Stk24,Synj2,Taf6,Tbx3,Tbx5,Tgfbr2,Tle3,Tox2,Trim63,Trmt11,U2af1l4,Ucp3,Uncx,Usp7,Zbtb42,Zbtb8a,Zdhhc22,Zfp37,Zfp90
**GO:0016188**	**synaptic vesicle maturation**	**3**	**8**	**0.049**	**Dlg4,Rab3a,Unc13a**
GO:0051129	negative regulation of cellular component organization	17	290	0.049	Arf6,Arrb2,Avp,Dbn1,Dlg4,Fzd9,Gfi1,Hgf,Inppl1,Mag,Mefv,Nrxn1,Pcsk9,Pif1,S1pr1,Shank1,Src

GO:0051252	regulation of RNA metabolic process	49	1219	0.049	Adora2a,Arrb2,Bmp6,Capn3,Cbx7,Cebpe,Chd6,Csdc2,Dapk3,Dnmt3a,Dpf1,Elavl2,Ezr,Fam129b,Fbxl15,Foxg1,Galr3,Gata2,Gdf6,Gfi1,Hnf1a,Kdm8,Map2k1,Mbnl2,Mical2,Myc,Nfe2,Nolc1,Nr3c1,Pax8,Phox2a,Pou2f1,Rere,Rnf187,S1pr1,Sbno1,Src,Taf6,Tbx3,Tbx5,Tgfb3,Tle3,Tox2,Uncx,Usp7,Zbtb42,Zbtb8a,Zfp37,Zfp90
GO:0098771	inorganic ion homeostasis	21	395	0.049	Ank3,Atp1a3,Avp,Bmp6,Cacna1a,Cacna1g,Cacna1i,Cacna2d1,Capn3,Dlg4,Fgfr4,Gata2,Gpr157,Gpr6,Grina,Itpr3,Jak3,Myc,S1pr1,Stim1,Trpm2
GO:2000641	regulation of early endosome to late endosome transport	3	8	0.049	Ezr,Map2k1,Src

Supplementary Table 3. Significant Biological Process GO terms for the seven genes of interest in this study.					
**#term ID**	**term description**	**observed gene count**	**background gene count**	**false discovery rate**	**matching proteins in your network (labels)**
**GO:0035176**	**social behavior**	**5**	**50**	**1.70E-09**	**DLG4,GRID1,NRXN1,NRXN3,SHANK1**
**GO:0071625**	**vocalization behavior**	**4**	**16**	**3.87E-09**	**DLG4,NRXN1,NRXN3,SHANK1**
**GO:0007612**	**learning**	**4**	**137**	**9.93E-06**	**DLG4,NRXN1,NRXN3,SHANK1**
GO:2000463	positive regulation of excitatory postsynaptic potential	3	26	9.93E-06	DLG4,NRXN1,SHANK1
GO:0035418	protein localization to synapse	3	40	2.32E-05	DLG4,NRXN1,SHANK1
GO:1900449	regulation of glutamate receptor signaling pathway	3	41	2.32E-05	DLG4,NRXN1,SHANK1
GO:0099601	regulation of neurotransmitter receptor activity	3	47	2.77E-05	DLG4,NRXN1,SHANK1
GO:0050885	neuromuscular process controlling balance	3	49	2.86E-05	DLG4,NRXN1,SHANK1
GO:0099173	postsynapse organization	3	54	3.28E-05	DLG4,NRXN1,SHANK1
GO:2000821	regulation of grooming behavior	2	5	7.56E-05	DLG4,NRXN1
GO:0072578	neurotransmitter-gated ion	2	7	0.00012	DLG4,NRXN1
GO:0007268	chemical synaptic transmission	4	402	0.00017	DLG4,GRID1,NRXN1,SYT3
GO:0042391	regulation of membrane potential	4	408	0.00017	DLG4,GRID1,NRXN1,SHANK1
GO:0048667	cell morphogenesis involved in neuron differentiation	4	400	0.00017	DLG4,NRXN1,NRXN3,SHANK1
GO:0060074	synapse maturation	2	9	0.00017	NRXN1,SHANK1
GO:0048812	neuron projection morphogenesis	4	448	0.00019	DLG4,NRXN1,NRXN3,SHANK1
GO:0007158	neuron cell-cell adhesion	2	14	0.00021	NRXN1,NRXN3
GO:0030534	adult behavior	3	137	0.00021	NRXN1,NRXN3,SHANK1
GO:0051962	positive regulation of nervous system development	4	488	0.00021	DLG4,NRXN1,SHANK1,SYT3
GO:0060997	dendritic spine morphogenesis	2	16	0.00025	DLG4,SHANK1
GO:2001257	regulation of cation channel activity	3	152	0.00025	DLG4,NRXN1,SHANK1
GO:0050877	nervous system process	5	1271	0.00031	DLG4,GRID1,NRXN1,NRXN3,SHANK1
GO:2000310	regulation of NMDA receptor activity	2	20	0.00035	DLG4,NRXN1
GO:2000311	regulation of AMPA receptor activity	2	20	0.00035	NRXN1,SHANK1
GO:0120035	regulation of plasma membrane bounded cell projection organization	4	600	0.00039	DLG4,NRXN1,SHANK1,SYT3

GO:0097120	receptor localization to synapse	2	23	0.0004	DLG4,NRXN1
GO:0001941	postsynaptic membrane organization	2	24	0.00041	DLG4,NRXN1
GO:0048699	generation of neurons	5	1422	0.00043	DLG4,NRXN1,NRXN3,SHANK1,SYT3
GO:0021700	developmental maturation	3	216	0.00051	DLG4,NRXN1,SHANK1
GO:0010976	positive regulation of neuron projection development	3	251	0.0007	DLG4,SHANK1,SYT3
GO:0051130	positive regulation of cellular component organization	4	1128	0.0025	DLG4,NRXN1,SHANK1,SYT3
GO:0097479	synaptic vesicle localization	2	92	0.0032	NRXN1,SYT3
GO:0007269	neurotransmitter secretion	2	95	0.0033	NRXN1,SYT3
GO:0099504	synaptic vesicle cycle	2	100	0.0036	NRXN1,SYT3
GO:0051129	negative regulation of cellular component organization	3	632	0.0063	DLG4,NRXN1,SHANK1
GO:0071248	cellular response to metal ion	2	162	0.0082	DLG4,NRXN1
GO:0061024	membrane organization	3	729	0.0091	DLG4,NRXN1,SYT3
GO:0023052	signaling	6	5108	0.0095	DLG4,GRID1,LRRTM4,NRXN1,NRXN3,SYT3
GO:0051049	regulation of transport	4	1732	0.0095	DLG4,NRXN1,SHANK1,SYT3
GO:0007154	cell communication	6	5219	0.0104	DLG4,GRID1,LRRTM4,NRXN1,NRXN3,SYT3
GO:0045859	regulation of protein kinase activity	3	788	0.0107	DLG4,LRRTM4,NRXN1
GO:0007411	axon guidance	2	220	0.0132	NRXN1,NRXN3
GO:0065008	regulation of biological quality	5	3559	0.0144	DLG4,GRID1,NRXN1,SHANK1,SYT3
GO:0051641	cellular localization	4	2180	0.0191	DLG4,NRXN1,SHANK1,SYT3
GO:0001525	angiogenesis	2	297	0.0213	NRXN1,NRXN3
GO:0016050	vesicle organization	2	318	0.0239	DLG4,SYT3
GO:0042221	response to chemical	5	4153	0.0269	DLG4,LRRTM4,NRXN1,NRXN3,SHANK1
GO:0032501	multicellular organismal process	6	6507	0.0292	DLG4,GRID1,NRXN1,NRXN3,SHANK1,SYT3
GO:0034613	cellular protein localization	3	1367	0.0397	DLG4,NRXN1,SHANK1
GO:0022604	regulation of cell morphogenesis	2	442	0.0402	DLG4,SYT3
GO:0007165	signal transduction	5	4738	0.0437	DLG4,GRID1,LRRTM4,NRXN1,NRXN3
GO:0060627	regulation of vesicle-mediated transport	2	480	0.0454	DLG4,SYT3
GO:0050794	regulation of cellular process	7	10484	0.0467	DLG4,GRID1,LRRTM4,NRXN1,NRXN3,SHANK1,SYT3
GO:0065003	protein-containing complex assembly	3	1514	0.0469	DLG4,NRXN1,SHANK1
GO:0045860	positive regulation of protein kinase activity	2	517	0.049	DLG4,NRXN1
GO:0009966	regulation of signal transduction	4	3033	0.0492	DLG4,LRRTM4,NRXN1,SHANK1

Supplementary Table 4: Rat RRBS sequencing metrics.

USER ID	TOTAL READ NUMBER (read pairs)	MAPPING EFFICIENCY (%)	UNIQUE CpGs	AVG. CpG COVERAGE	BISULFITE CONVERSION RATE (%)
RAT-1T	27,790,873	25	2,227,723	17X	98
RAT-2C	25,837,561	22	2,187,473	14X	98
RAT-5C	23,882,262	24	2,086,635	15X	98
RAT-6C	26,339,515	23	2,207,304	15X	98
RAT-7T	26,620,354	25	2,291,358	16X	99
RAT-8C	28,111,616	24	2,304,172	17X	98
RAT-9T	25,104,000	23	2,188,362	15X	99
RAT-10T	26,355,902	22	2,104,074	16X	98
RAT-11C	26,787,715	19	1,888,084	17X	98
RAT-12C	27,634,936	23	2,204,322	16X	98
RAT-13C	28,557,777	31	2,427,768	19X	98
RAT-14C	29,019,936	33	2,504,894	20X	98
RAT-15T	23,085,177	31	2,327,653	16X	98
RAT-16T	23,692,161	31	2,295,185	17X	98
RAT-17C	27,598,160	29	2,368,799	18X	98
RAT-18T	27,537,998	31	2,352,778	19X	98
RAT-19T	25,783,780	24	1,932,160	19X	98

Summary of rat RRBS sequencing parameters. T denotes THC-exposed rats, C denotes control rat.
